# Genetic Connections and Convergent Evolution of Tropical Indigenous Peoples in Asia

**DOI:** 10.1093/molbev/msab361

**Published:** 2021-12-23

**Authors:** Lian Deng, Yuwen Pan, Yinan Wang, Hao Chen, Kai Yuan, Sihan Chen, Dongsheng Lu, Yan Lu, Siti Shuhada Mokhtar, Thuhairah Abdul Rahman, Boon-Peng Hoh, Shuhua Xu

**Affiliations:** 1 State Key Laboratory of Genetic Engineering, Center for Evolutionary Biology, Collaborative Innovation Center for Genetics and Development, School of Life Sciences, Fudan University, Shanghai, China; 2 Key Laboratory of Computational Biology, Shanghai Institute of Nutrition and Health, University of Chinese Academy of Sciences, Chinese Academy of Sciences, Shanghai, China; 3 Department of Liver Surgery and Transplantation Liver Cancer Institute, Zhongshan Hospital, Fudan University, Shanghai, China; 4 Ministry of Education Key Laboratory of Contemporary Anthropology, School of Life Sciences, Human Phenome Institute, Fudan University, Shanghai, China; 5 Institute of Medical Molecular Biotechnology, Faculty of Medicine, Universiti Teknologi MARA, Sungai Buloh Campus, Sungai Buloh, Selangor, Malaysia; 6 Clinical Pathology Diagnostic Centre Research Laboratory, Faculty of Medicine, Universiti Teknologi MARA, Sungai Buloh Campus, Sungai Buloh, Selangor, Malaysia; 7 Faculty of Medicine and Health Sciences, UCSI University, Cheras, Kuala Lumpur, Malaysia; 8 School of Life Science and Technology, ShanghaiTech University, Shanghai, China; 9 Jiangsu Key Laboratory of Phylogenomics and Comparative Genomics, School of Life Sciences, Jiangsu Normal University, Xuzhou, China; 10 Henan Institute of Medical and Pharmaceutical Sciences, Zhengzhou University, Zhengzhou, China; 11 Center for Excellence in Animal Evolution and Genetics, Chinese Academy of Sciences, Kunming, China

**Keywords:** tropical indigenous populations, population admixture, genetic diversity, local adaptation, skin pigmentation, convergent evolution

## Abstract

Tropical indigenous peoples in Asia (TIA) attract much attention for their unique appearance, whereas their genetic history and adaptive evolution remain mysteries. We conducted a comprehensive study to characterize the genetic distinction and connection of broad geographical TIAs. Despite the diverse genetic makeup and large interarea genetic differentiation between the TIA groups, we identified a basal Asian ancestry (bASN) specifically shared by these populations. The bASN ancestry was relatively enriched in ancient Asian human genomes dated as early as ∼50,000 years before the present and diminished in more recent history. Notably, the bASN ancestry is unlikely to be derived from archaic hominins. Instead, we suggest it may be better modeled as a survived lineage of the initial peopling of Asia. Shared adaptations inherited from the ancient Asian ancestry were detected among the TIA groups (e.g., *LIMS1* for hair morphology, and *COL24A1* for bone formation), and they are enriched in neurological functions either at an identical locus (e.g., *NKAIN3*), or different loci in an identical gene (e.g., *TENM4*). The bASN ancestry could also have formed the substrate of the genetic architecture of the dark pigmentation observed in the TIA peoples. We hypothesize that phenotypic convergence of the dark pigmentation in TIAs could have resulted from parallel (e.g., *DDB1/DAK*) or genetic convergence driven by admixture (e.g., *MTHFD1* and *RAD18*), new mutations (e.g., *STK11*), or notably purifying selection (e.g., *MC1R*). Our results provide new insights into the initial peopling of Asia and an advanced understanding of the phenotypic convergence of the TIA peoples.

## Introduction

Asia is home to approximately 370 million self-identified indigenous peoples, comprising two-thirds of the total indigenous population in the world. These populations are culturally and physiologically diverse and have been largely shaped by their local environment and subsistence. These unique characteristics offer an incredible opportunity to study human evolution and have long been a topic of interest and speculation. Archaeological records document a long history of modern human occupation in Asia, which probably occurred 50–70 ka along the coast of the Indian subcontinent to the Sundaland, subsequently reaching the Sahul Shelf (Bae et al. 2017). Several rainforest tribal populations scattered throughout the tropical areas of Asia and Oceania are thought to be descended from the original inhabitants of Asia, including the Melanesians, the Aboriginal Australians, and the Negritos currently found in the Andaman Islands, Peninsular Malaysia, and the Philippine Islands (Bae et al. 2017). This hypothesis was first proposed based on morphological features of these people and has received substantial attention in recent years due to mounting evidence from anthropologic and genetic studies (Oppenheimer 2009; Malaspinas et al. 2016; Mondal et al. 2016; Skoglund et al. 2016; Yelmen et al. 2019; Wang et al. 2021). The arrival of Austroasiatic and Austronesian farmers from the north during the Late Paleolithic and Neolithic era forced the early inhabitants into separate areas, which added to the complex demographic history of these peoples (Bellwood 2007; Jinam et al. 2012; Lipson et al. 2014). Many genetic studies have highlighted substantial distinction among geographically separated indigenous populations for their distinct genetic affinities and admixture patterns (Endicott et al. 2003; Reich et al. 2010, 2011; Jinam et al. 2017; GenomeAsia100K Consortium 2019), but only a few have characterized the genomic footprints of their possibly shared evolution (Hoh et al. 2020).

One question that has long been debated is whether the distinctive physical features (e.g., small body size, dark skin, curly hair, and broad nose, collectively known as “Negrito-like” phenotypes) shared by the tropical hunter-gatherers are ancestral or derived as a result of genetic convergence. Migliano et al. (2013) identified various signals of growth-related positive selection in different tropical indigenous populations, whereas Bergey et al. (2018) reported shared natural selections on height-related pathways in African and Asian rainforest hunter-gatherers. Relatively more analyses were carried out in single tropical indigenous populations or geographically related indigenous groups. Novel adaptive signals of height, hair, nasal morphology, and those in response to some extreme environmental stresses have been reported in each of these populations or groups (Liu et al. 2015; Lopez et al. 2019; Zhang et al. 2021). However, the current understanding of the genetic basis of the dark skin phenotype is rather limited. Over the past few decades, tremendous advances have been made in our understanding of the evolutionary history of light skin in Europeans and East Asians, whereas only a few recent studies revealed the skin color variations and related loci in populations of African ancestry (Bonilla et al. 2005; Beleza et al. 2013; Crawford et al. 2017; Hernandez-Pacheco et al. 2017; Lloyd-Jones et al. 2017; Martin et al. 2017). Interestingly, some of the dark pigmentation-associated variants could have been under parallel evolution in different continents (Crawford et al. 2017). An alternative argument is that dark skin, as well as other “Negrito-like” phenotypes, are probably products of convergent adaptations, as evidenced by extensive genetic differentiation among these populations (Endicott et al. 2003; GenomeAsia100K Consortium 2019). Although exposed to intensive ultraviolet (UV) radiation all year round at similar latitudes, the indigenous populations are more darkly pigmented than their counterparts. This was evidenced by visual impressions and reported melanin measures (demonstrated in supplementary note S1, Supplementary Material online). The long history and simple modes of subsistence (e.g., foraging, pastoralism, and horticulture) of these populations would give special insights into the genetic mechanism of human pigmentation evolution.

Efforts have been made to investigate the full landscape of human genomic diversity in Asia (HUGO Pan-Asian SNP Consortium 2009; GenomeAsia100K Consortium 2019), and the importance of genetic data of minority groups in understanding human evolution and disease has been highlighted (Ben-Eghan et al. 2020). However, the lack of comprehensive geographical samples and full characterization of the genomic variants have limited our understanding of the genetic relationship and local adaptations of the tropical indigenous Asian (hereafter abbreviated as TIA) populations. In this study, we aim to explore the genetic connections of the TIA populations that share similar phenotypes, and further illustrate the putative genetic mechanisms underlying their phenotypic convergence. We compiled and analyzed genome-wide single-nucleotide polymorphism (SNP) data of 2,845 sequenced samples and 3,214 genotyped samples from global populations. These data provide especially high geographical coverage for the TIA populations that can be contextualized using very dense non-TIA samples, including tropical samples from Africa and the Americas, and a wide range of nonindigenous samples in Asia (supplementary fig. S1*A*–*C*, Supplementary Material online). The TIA populations were further split into two subcategories—one is the anthropologically identified Negrito with the above-mentioned outstanding aboriginal phenotypes, denoted herein as Negrito-like TIA (NL-TIA); the other includes the non-Negrito-like TIA (nNL-TIA) populations. Detailed information on the samples and the groupings can be found in supplementary note S2, Supplementary Material online. We note that the Oceanic populations, although not located in the Asia continent, were also included in our analyses as a group of TIAs, considering that Southeast Asia and the Pacific islands are tightly related in modern human migration history (Gibbons 2001; Xu et al. 2012). We investigated the genetic relationship and ancestry makeup of the TIA populations, based on which we characterized the natural selections by focusing on the genetic architecture of previously identified pigmentation-related loci. We further explored the adaptive hypothesis of convergent evolution by evaluating these candidates, and finally developed a comprehensive model for the evolution of the dark skin phenotype in different geographical TIA populations.

## Results

### Genetic Affinity and Distinction of the TIA Populations

**Fig. 1.**
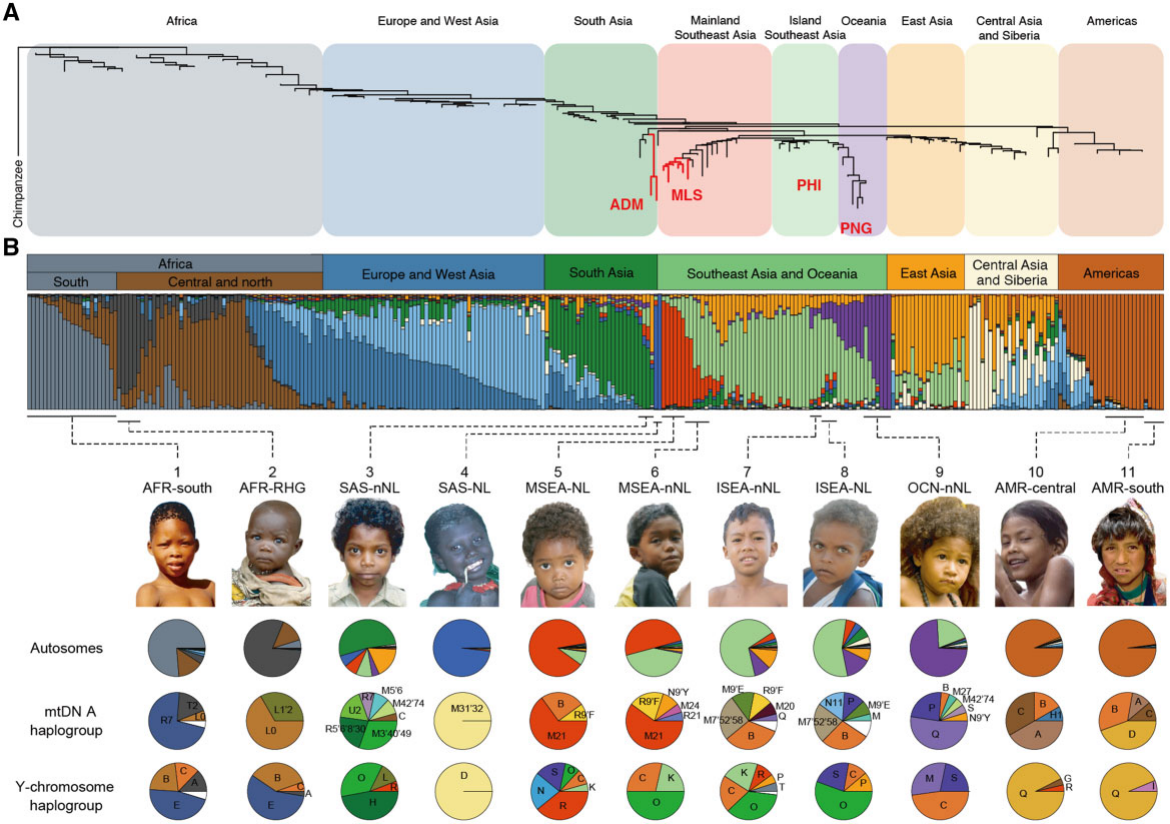
Population phylogeny and ancestry inference of worldwide tropical indigenous groups. (*A*) A population neighbor-joining tree constructed based on *F*_ST_ using 49,489 genome-wide SNPs. The Chimpanzee genome was used as an outgroup. The Negrito-like (NL) tropical indigenous populations in Asia (TIA) are highlighted with thick red branches. (*B*) The ancestry components were inferred based on autosomal SNPs, mtDNA, and Y-chromosome data. The autosome-based inference is provided by ADMIXTURE analysis at *K* = 13. The mtDNA and Y haplogroups for some populations were obtained from the literature, whereas those for the others were estimated from the SNP-array or NGS data analyzed in this study (see supplementary table S1, Supplementary Material online, for more details). One portrait is shown for each group of tropical indigenous peoples, representing 1, San from southern Africa; 2, Batwa from central Africa; 3, Irula from mainland India; 4, Jarawa from the Andaman Islands; 5, Bateq from Peninsular Malaysia; 6, CheWong from Peninsular Malaysia; 7, Bajo from Indonesia 8, Aeta from the Philippines; 9, Papuan from Papua New Guinea; 10, Ticuna from Central America; 11, Quechua from South America. Photo sources: https://www.gettyimages.in/ (last accessed December 29, 2021) for 1, 2, 4, and 8–11; https://www.pinterest.com (last accessed December 29, 2021) for 3; https://www.vladivlad.com (last accessed December 29, 2021) for 7; Photos 5 and 6 were taken from the fieldwork. NL; Negrito-like; nNL, non-Negrito-like; AFR, Africa; SAS, South Asia; MSEA, mainland Southeast Asia; ISEA, island Southeast Asia; OCN, Oceania; EAS, East Asia; AMR, the Americas.

Tropical indigenous populations are highly structured in genetics, consistent with their geographic relatedness (fig. 1 and supplementary fig. S1*D* and *E*, Supplementary Material online). The TIAs did not show any observable connections with their counterparts from Africa and the Americas (fig. 1 and supplementary figs. S1 and S2, Supplementary Material online). Principal component analysis (PCA) showed that they formed distinct clusters along the longitude on PC1 and largely corresponded to the Asia mainland and the Oceanic islands on PC2 (supplementary fig. S1*E*, Supplementary Material online). TIAs from South Asia and Oceania were more widely distributed along PC1 than those from Southeast Asia. The NL-TIAs from the Andaman Islands (ADM), Malaysia (MLS), and the Philippines (PHI) compacted against the long stretches of the Oceanic populations, but were distinguished after we removed all the other populations (supplementary fig. S1*F*, Supplementary Material online). We observed that the largest population differentiation (measured by *F*_ST_) in Asia was among the NL-TIA populations (i.e., ADM, MLS, and PHI), and the Papuans (PNG) (fig. 1*A*), and was enriched in genes involved in olfactory transduction (hsa04740; false discovery rate-corrected *P*=0.2 × 10^−4^ by clusterProfiler; Yu et al. 2012).

Despite the fact that most of the global populations have multiple sources of genetic components, ADM, MLS, and the Oceanic populations contributed three additional ancestry components and showed minimal evidence of recent admixture (fig. 1*B*). This result is consistent with the small effective population size and long identical-by-descent (IBD) tracts of these populations (supplementary fig. S3, Supplementary Material online). The ancestry makeup of these three populations was more homogeneous than the African rainforest hunter-gatherers, who are also dominated by a group-specific genetic component. However, PHI was characterized as admixed descendants of the Oceanic populations and the Austronesians (fig. 1*B*). The nNL tropical indigenous populations from South Asia, island Southeast Asia, and the Americas showed similar patterns of ancestry makeup with the nonindigenous populations in these three regions, respectively, and were less likely to be affected by the NL-TIAs.

Consistent with the autosomal genetic pattern, mitochondrial DNA (mtDNA) haplogroups were generally restricted in local regions. Populations with shared genetic ancestry may share the same mtDNA haplogroups (fig. 1*B* and supplementary table S1, Supplementary Material online). For instance, haplogroup M21, which possibly represents the ancient maternal components in Southeast Asia (Macaulay et al. 2005; Hill et al. 2006, 2007), is the major haplogroup in NL- (65%) and nNL-TIAs (60%) of mainland Southeast Asia; Haplogroup P, which is commonly found in Oceania (Friedlaender et al. 2005), was identified in PNG (24%) and PHI (12%). The Y-chromosome data confirmed the cross-continent barrier but showed some levels of interaction among populations within the continent (fig. 1*B* and supplementary table S1, Supplementary Material online). For example, the haplogroup S, which was thought to be centered on the highlands of Papua New Guinea, was identified in several males of PHI and MLS. Although different subtypes of haplogroup S were assigned to each region (S1d and S1a in PNG, S3a in PHI, and unspecified types noted as S* in MLS), they could have been inherited from a common paternal lineage dated back to 48 ka. In addition, ADM shared the D1 haplogroups with Tibetans (supplementary table S1, Supplementary Material online), a group of highland indigenous peoples neighboring South Asians. These results collectively suggest sex-biased and ancestry-biased human migration and admixture history of the TIA populations.

### A Shared Genetic Ancestry of the TIA Populations

**Fig. 2.**
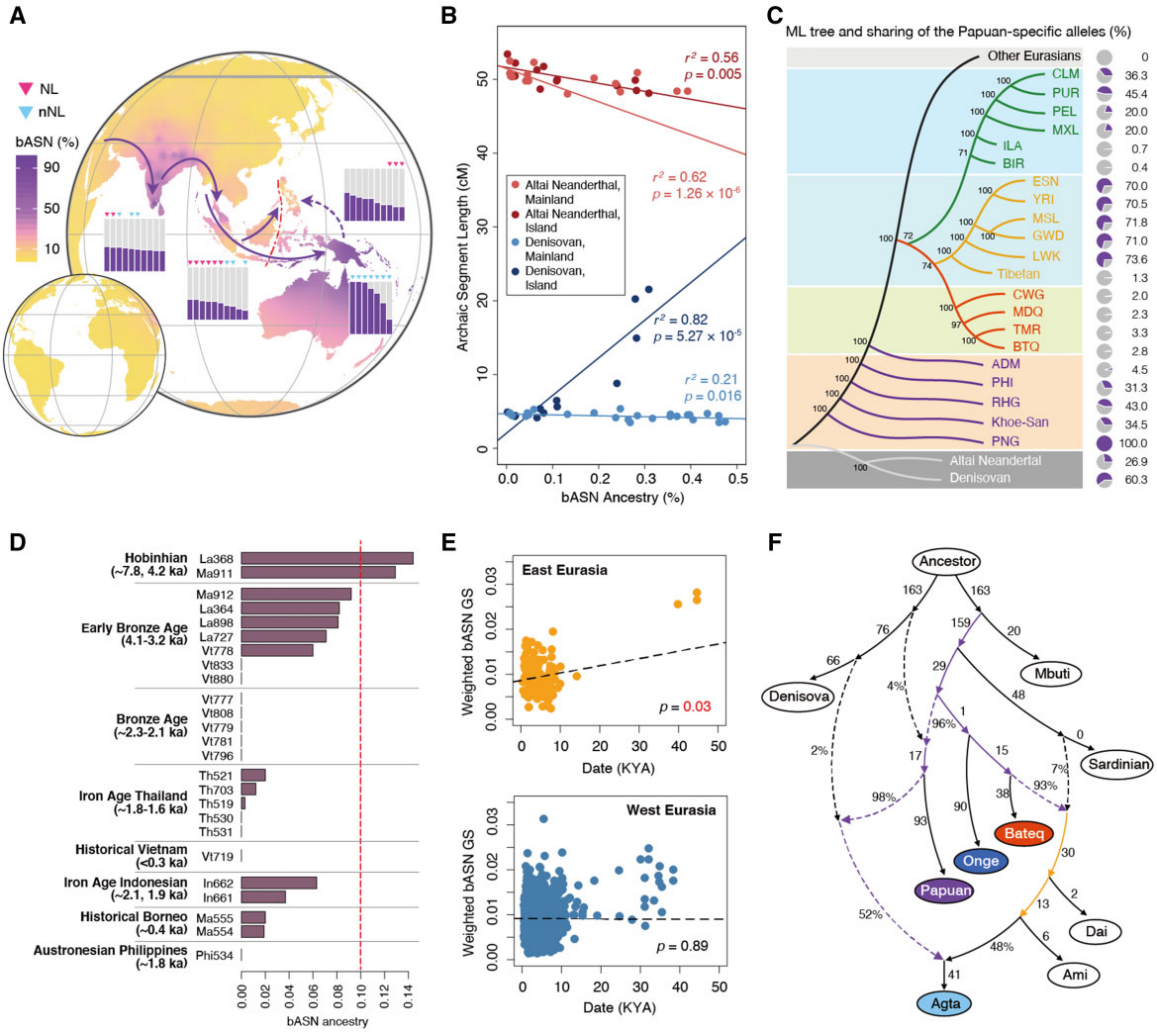
Evidence for the basal Asian (bASN) ancestry. (*A*) Global distribution of the bASN ancestry, inferred by the ADMIXTURE analysis at *K* = 5. The purple arrows denote the possible route of the independent pulse of early migration to Asia. The bar plots show the bASN component proportions across populations. Populations are sorted according to the bASN proportions, and only the top ten populations are presented for each region. (*B*) Correlation between lengths of the introgressed segments from the archaic hominins and proportion of the bASN ancestry in Asian populations. The mainland populations include South Asians, mainland Southeast Asians, and East Asians; the island populations include island Southeast Asians. (*C*) Maximum-likelihood tree for the Papuan (PNG)-specific loci and sharing of the PNG-specific alleles (%). (*D*) Inferred bASN ancestry in Neolithic Southeast Asian samples when they were projected in the ADMIXTURE analysis. The red dashed line indicates the bASN ancestry level of 0.1. (*E*) GS of the bASN alleles in Neolithic-Paleolithic Eurasian samples. (*F*) An admixture graph fitting the indigenous genomes within the broader scenario of global population splits and subsequent admixtures. The purple arrows indicate the path for the bASN ancestry, and the yellow arrows indicate the modern East Asian ancestry. Lengths and admixture proportions are shown for the branches. RHG, rainforest hunter-gatherers in Africa; PHI, Philippine Negrito; ADM, Andaman Negrito; BTQ, Bateq Negrito; TMR, Temiar; MDQ, Mendriq Negrito; CWG, CheWong; BIR, Birhor; ILA, Irula. Other modern human populations are from the 1000 Genomes Project data set.

The intrinsic genetic connections among the TIA populations indicated by the Y chromosome were further consolidated on the autosomes by the unsupervised ADMIXTURE analysis at *K* = 5, where we found one basal component specifically prevailing in Asia, with leading proportions in the Oceanic populations, followed by some South and Southeast Asian populations (fig. 2*A* and supplementary fig. S4*A*, Supplementary Material online). Interestingly, this ancestry was substantially enriched in the TIA populations, especially in the NL-TIAs. This result was supported by eight of the ten replication analyses of ADMIXTURE with random seeds (supplementary fig. S4*B*, Supplementary Material online). The basal component is unlikely a result of random genetic drift, which may have large effects on the isolated populations but would casually shape them to different directions; nor is it likely driven by recent gene flow, as it is enriched in geographically discrete TIA populations that are largely hindered by geographical discontinuity.

We speculate this basal Asian (bASN) ancestry to be an ancient lineage, as evident by the following. First, the bASN ancestry is negatively correlated with the Altai Neanderthal introgression in Asian populations (*r*^2^=0.62 and *P* = 1.26 × 10^−6^ for the mainland populations; *r*^2^=0.56 and *P* = 0.005 for the island populations), suggesting that the peopling of Asia might have occurred prior to the Altai Neanderthal introgression (directly or indirectly) to the modern human populations in this region (fig. 2*B*). Second, the bASN-specific alleles (represented by the Papuan-specific alleles) possibly originated from the ancient gene pool of the modern human ancestry, as they share a deep root with the Africans and archaic hominins on the maximum-likelihood tree (fig. 2*C*), in contrast to those specific to the European and East Asian ancestries (represented by present-day Europeans and East Asians, respectively) (supplementary fig. S5, Supplementary Material online). Moreover, we recapitulated this shared ancestry in some ancient genomes yielded from Neolithic Southeast Asians when they were projected in the ADMIXTURE analysis (fig. 2*D*). Intriguingly, the bASN ancestry is centered in the Indo-China Peninsula, and is positively correlated with time (*r*^2^=0.35, *P* = 0.001; supplementary fig. S6, Supplementary Material online). We then examined the sharing of 14,353 bASN-derived alleles, which were identified from the bASN-enriched TIA populations (i.e., ADM, MLS, PHI, and PNG) and were representative of the bASN ancestry (see Materials and Methods, supplementary fig. S7*A*, Supplementary Material online), in global Neolithic-to-Paleolithic human samples (0–50,000 ka). We found a significant positive correlation between the time and genetic score (GS) of bASN-derived alleles in East Eurasian samples (*P* = 0.03), but this was not observed in West Eurasian samples (*P* = 0.89) (fig. 2*E* and supplementary fig. S7*B*, Supplementary Material online). The bASN-derived alleles were also relatively enriched in the Denisovan sequence (Reich et al. 2010) (fig. 2*C*), but this ancestry cannot be fully explained by a shared event of archaic introgression in the TIAs, as >80% of the bASN-derived alleles were not found in the Denisovan introgressed segments (supplementary fig. S7*C*, Supplementary Material online). The significant positive correlation between bASN ancestry and Denisovan introgression in the island Southeast Asians (*r*^2^=0.82; *P* = 5.27 × 10^−5^) (fig. 2*B*) possibly reflects recent gene flow cross the Wallace–Huxley’s line, which is indicated by the TreeMix analysis (fig. 2*A* and supplementary fig. S8, Supplementary Material online).

MixMapper (Lipson et al. 2014) fit all the tested TIA populations, including the Melanesians from Bougainville, PHI, Malaysian Orang Asli, and ADM, as combinations of two ancestry components: one closely related to PNG branch (representing the bASN ancestry); another split deeply from the East Asians (supplementary fig. S9*A*, Supplementary Material online). Intriguingly, the relative proportions are highly correlated with those inferred using ADMIXTURE (supplementary fig. S9*B*, Supplementary Material online). Admixture graphs constructed using AdmixtureBayes (Nielsen 2018) and qpGraph (Patterson et al. 2012) showed that PNG, ADM, and MLS received ancestries that split deeply with Europeans (fig. 2*F*), and they contributed greatly to their surrounding nNL-TIAs and nonindigenous populations in Asia. For the South Asian groups, the Onge-related ancestor contributes 80% ancestry to Irula (nNL) and 48% ancestry to Gujarati (nonindigenous), with the remaining ancestry contributed by the European ancestor (supplementary fig. S10*A*, Supplementary Material online); for groups in Malaysia, the Negrito ancestor contributes 59% to Temiar (nNL) and a lesser extent to Malay (nonindigenous) (supplementary fig. S10*B*, Supplementary Material online). However, PHI genomes were almost equally contributed by PNG and the nonindigenous populations in East Asia, possibly due to replacement by Austronesian expansion (supplementary fig. S10*C*, Supplementary Material online). This observation is in agreement with the genetic makeup of PHI inferred by ADMIXTURE. These collective findings therefore strongly support the basal role of the indigenous populations in modern human migration to Asia (fig. 2*A*).

### Shared Adaptations of the TIA Populations

Given the wide range of geographical TIA populations studied and the shared bASN ancestry identified, we intended to trace the footprints of this common ancestry and explore its possible functional effects on present-day TIA populations. We estimated the density of the bASN-affected SNPs and the frequency of the bASN-derived haplotypes along the genome, and found some genomic regions were enriched with the bASN ancestry, and thus could be regarded as putative candidates of bASN-related adaptations (supplementary table S2, Supplementary Material online). We focused on the shared adaptations in at least two out of the four representative TIA populations, which implies possible parallel or convergent evolution (table 1). Although some other TIA populations also showed enriched bASN ancestry, the sample size for these populations was insufficient (<5 samples for each) for unbiased frequency estimation. One of the outstanding examples is a bASN-derived haplotype in *LIMS1*. It consists of 40 bASN alleles spanning the entire gene (∼150 kb), all of which are derived alleles. The frequency of this haplotype is 0.97 in PNG, 0.8 in ADM, <0.3 in MLS and PHI, and is very rare in other worldwide populations. The high frequency and long stretch of this haplotype suggested that it could be adaptive in PNG and ADM (supplementary fig. S11, Supplementary Material online). *LIMS1* encodes an adaptor protein that plays a role in integrin-mediated cell adhesion or spreading. It has also been reported to be hairiness-associated in East Asians (Endo et al. 2018). Downstream to *LIMS1* is *EDAR*, a well-recognized gene that is responsible for the facial and hair features of East Asians (Adhikari et al. 2016; Wu et al. 2016). The Asian prevalent allele at the key missense variant in *EDAR*, rs3827760-G, appears in lower frequency in the Southeast Asian indigenous populations (frequency=0.25–0.44), but did not show signals of adaptation in any of these populations. We also identified a group of candidate haplotypes (∼51 kb) in *COL24A1*, the frequency of which is higher in PNG (0.47), MLS (0.31), Vellalar (an nNL-TIA population in South Asia; 0.28), CheWong (an nNL-TIA population in Southeast Asia; 0.25), Tibetan (0.20), and African populations (0.25–0.42) than the rest of populations studied. *COL24A1* encodes for collagen XXIV predominantly functioning in osteoblast differentiation and bone formation and is associated with some morphological traits in humans, such as waist–hip ratio and height (Lotta et al. 2018; Kichaev et al. 2019). More interestingly, a 56.9-kb adaptive haplotype in *DDB1*/*DAK* was identified in ADM (0.95), PHI (0.5), and African populations (0.52–0.83), and it included a key missense variant (rs2260655, c.553G>A, p. A185T) associated with the melanin index of Africans (Crawford et al. 2017). This observation supports the shared evolution of skin pigmentation between African and Asian populations proposed by Crawford et al. (2017), by complementing the hypothesis with more significant evidence via the understudied tropical indigenous populations in South and Southeast Asia. Other shared signatures of selection in several TIA populations include *LRBA* aiding the secretion and membrane deposition of immune effector molecules, *NRXN3* encoding neurexins that function in the vertebrate nervous system as cell adhesion molecules and receptors, and several genes involved in the metabolic pathways (e.g., *ACBD6*, *FDXR*, *INPP4B*, and *SH3PXD2A*) (table 1).

**Table 1.** Top 20 bASN-Enriched Genomic Regions Showing Shared Adaptation among TIA Populations.

No.	Chr	Region (Mb)	No. Total SNPs	No. bASN SNPs	% bASN SNPs	Protein-Coding Genes	ADM			MLS			PHI			PNG		
							No. SNPs	*f*_mean_	*f*_max_	No. SNPs	*f*_mean_	*f*_max_	No. SNPs	*f*_mean_	*f*_max_	No. SNPs	*f*_mean_	*f*_max_
1	13	53.25–53.3	280	42	15.0	*SUGT1, LECT1*	—	—	—	42	0.28	0.28	—	—	—	42	0.27	0.27
2	4	151.55–151.6	189	28	14.8	*LRBA*	28	0.40	0.40	28	0.31	0.31	28	0.28	0.28	28	0.43	0.43
3	1	86.2–86.25	254	34	13.4	*COL24A1*	—	—	—	34	0.31	0.31	—	—	—	33	0.36	0.47
4	15	37.05–37.1	347	40	11.5	*C15orf41*	31	0.23	0.25	—	—	—	—	—	—	40	0.43	0.43
5	1	180.4–180.45	302	34	11.3	*ACBD6*	2	0.43	—	31	0.22	0.22	32	0.31	0.33	32	1.00	1.00
6	13	53.2–53.25	267	29	10.9	*SUGT1, HNRNPA1L2*	—	—	—	29	0.28	0.28	—	—	—	29	0.27	0.27
7	4	142.95–143	329	30	9.1	*INPP4B*	—	—	—	5	0.28	0.28	30	0.29	0.33	30	0.27	0.27
8	1	61.6–61.65	253	23	9.1	*NFIA*	7	0.20	0.20	23	0.44	0.44	—	—	—	—	—	—
9	15	57.95–58	332	29	8.7	*GCOM1, MYZAP, POLR2M*	—	—	—	29	0.25	0.25	—	—	—	26	0.52	0.60
10	2	109.15–109.2	305	25	8.2	*LIMS1*	25	0.80	0.80	25	0.28	0.28	25	0.22	0.22	25	0.97	0.97
11	10	105.6–105.65	313	25	8.0	*OBFC1, SH3PXD2A*	25	0.45	0.45	—	—	—	2	0.22	—	25	0.56	0.57
12	1	180.35–180.4	327	26	8.0	*ACBD6*	2	0.45	—	24	0.23	0.22	24	0.32	0.33	24	1.00	1.00
13	1	180.45–180.5	306	24	7.8	*ACBD6*	3	0.42	—	21	0.22	0.22	21	0.31	0.33	21	1.00	1.00
14	11	61.1–61.15	217	16	7.4	*DDB1, DAK, CYB561A3, TMEM138*	16	0.96	0.95	1	0.25	—	16	0.51	0.50	4	0.20	—
15	17	72.85–72.9	383	28	7.3	*GRIN2C, FADS6, FDXR*	28	0.21	0.20	—	—	—	—	—	—	28	0.20	0.20
16	14	79.55–79.6	290	21	7.2	*NRXN3*	1	0.20	—	21	0.33	0.33	1	0.33	—	21	0.23	0.23
17	7	158.15–158.2	444	32	7.2	*PTPRN2*	—	—	—	32	0.23	0.25	—	—	—	5	0.30	0.30
18	11	61.05–61.1	222	16	7.2	*DDB1, VWCE*	16	0.95	0.95	—	—	—	16	0.51	0.50	—	—	—
19	12	71.95–72	352	25	7.1	*LGR5*	25	0.25	0.25	—	—	—	—	—	—	25	0.27	0.27
20	6	168.9–168.95	493	34	6.9	*SMOC2*	34	0.41	0.45	—	—	—	1	0.22	—	5	0.27	0.27

Note.—*f*_mean_, average bASN-derived AF; *f*_max_, maximum bASN-derived AF, obtained from the bASN-AF block consisting of at least five bASN-derived alleles; ADM, Andaman Negrito; MLS, Malaysian Negrito; PHI, Philippine Negrito; PNG, Papuan.

We alternatively employed a de novo screening strategy to search for more shared selective sweeps, by estimating a composite statistic called *F*_CS_ based on the integrated haplotype score (iHS) and locus-specific branch length (LSBL) (see Materials and Methods). Remarkably, we identified two genomic regions that exhibited sweep signals in all four populations (supplementary fig. S12*A*, Supplementary Material online). One of these regions lies in *NKAIN3*, which encodes a member of the Na^+^/K^+^-ATPase β-subunit interacting protein family that is involved in the regulation of the Na^+^ transport (supplementary fig. S12*B*, Supplementary Material online). This gene is specifically expressed in the brain and nerves according to the Genotype-Tissue Expression (GTEx) database (https://gtexportal.org/home/, last accessed December 29, 2021) and is associated with certain neurological diseases. Notably, it has been reported to be height-associated in the Europeans (Tachmazidou et al. 2017). The other signal is in *C16orf97*, an lncRNA gene. GTEx shows that this gene expresses significantly in testis as opposed to other organs. We also found that several genes showed different adaptive loci among the four populations. One example is *TENM4*, which encodes the teneurin transmembrane protein 4 that plays a role in establishing proper neuronal connectivity during development (supplementary fig. S12*C*, Supplementary Material online). Adaptive genes shared by at least two of the four populations were significantly enriched in neurological functions (supplementary fig. S12*D*, Supplementary Material online). There was also evidence for shared adaptation on the Negrito-like phenotypes, attributing to identical candidate genes across TIA populations or different genes for similar biological processes (table 2). Notably, there were a larger number of candidate genes shared across the Wallace–Huxley’s line (between ADM/MLS and PHI/PNG), than those identified specifically in population grouping located at either side (within ADM/MLS or PHI/PNG), suggesting that those shared signals are unlikely to be consequences of recent gene flow between populations. These results indicate complex genetic mechanisms underlying the phenotypic convergence of TIA populations.

**Table 2.** Putative Signals of Local Adaptation Related to the “Negrito-Like” Phenotypes in TIA Populations.

Trait	ADM	MLS	PHI	PNG
Height	MKL1*, NUCB2*, ZBTB24*, RP11-219B17.1*, RORA*, SEPT2*,FER*, TTLL5*, PDE11A*, TEAD1*, DYM*, *UQCC1*, *PLAG1*, *CEP250*, *ZFAS1*, *GDF5**, HHIP, ITIH3, MICAL1, CCDC91, RP11-110H1.9, RP11-326A19.4, PLEKHA1, EXTL3, ZNF438, STAU1, VGLL4, TRIP11, MYO9A, SLIT3, AMZ1, ZNF367, HHIP-AS1, MAP3K3, PEMT, TMED10, FILIP1, TNRC6B, PPIL6, PTPN14, VTA1, GNA12, LCORL*	ATAD5*, MEF2C-AS1*, SCMH1*, AC007091.1*, MAGI2*, CTD-2349P21.11*, DLG2*, EDEM2*, *UQCC1*, *PLAG1*, *CEP250*, *ZFAS1*, *GDF5**, ANKS1A, ERC1, DCLK1, CTU2, PPARD, COLGALT2, H6PD, GLI2, FADS2, ZBTB7C, ZNF678, ETV6, FADS1, ZNF76, FGF18, PAPPA2, RAD51B, TSEN15*	ATAD5*, NUCB2*, ZBTB24*, MEF2C-AS1*, SCMH1*, FER*, TTLL5*, PDE11A*, CTD-2349P21.11*, TEAD1*, DLG2*, EDEM2*, *LPAR1*, *PHF21A*, *DNM3*, *CDH13*, *PRKCA*, *SUCLG2**, ARHGAP24, NSD1, PSORS1C1, SLC38A9, RSBN1L-AS1, PPP2R2A, CLOCK, RP11-362A1.1, NUP37, RSRC1, ADAP2, SYN3*	MKL1*, RP11-219B17.1*, RORA*, SEPT2*, AC007091.1*, MAGI2*, DYM*, *LPAR1*, *PHF21A*, *DNM3*, *CDH13*, *PRKCA*, *SUCLG2*, *SYTL2, FSTL5, PASK, SLBP, RP1-35C21.2, LRRC37B, RP11-977G19.10, CS, SBNO1, DLEU1, ZFAT, DOCK3, SERPINA1, INSR, TTLL4, IPPK, ANTXR1, ZNF142, PVT1, HMGA2*
Sitting height ratio	TEAD1*	JAK1*, PTPRM, EXOC2	TEAD1*, ATAD2B, UBXN2A	JAK1*, EPHB1, CTB-33O18.1
Hair color: black	HNRNPA1P36*, SNX16*, MARK3*, RP13-923O23.6*, *ERMP1*, *FRAS1*, *KIAA2026**, RP11-556I14.2, VGLL4, PTAFR, ATP1B3, PEX14, TCF20, NTM*	GPR98*, *ERMP1*, *FRAS1*, *KIAA2026**, AL163953.3, TMEM258, RP11-459E5.1, EXOC2, IL16, PEBP4, GAB2, IRF4, RP11-452H21.1, MED13L, CDC42BPA, RAD51B*	HNRNPA1P36*, SNX16*, RP13-923O23.6*, *AC092614.2**, CTD-2081C10.1, RSPO2, ARHGAP24, SOX6, C4orf22, GFM2, HEXB*	GPR98*, MARK3*, *AC092614.2**, LHX2, AC003958.6, CHL1, MITF, RPL34-AS1, NFIA, KRT31, ZNF831*
Hair shape	PTK6*, *FRAS1*	*FRAS1*	PTK6*	PTK6*
Skin pigmentation	TUSC3*, AC016682.1, AC113610.1, PRTFDC1	*MEGF11, IRF4*	*RNF111, SLFN12L*	TUSC3*, LINC00504, PLEKHA6
Skin reflectance	*RP11-184D12.1*	*TNFRSF10B, RIMBP2*	*RP11-6N13.1* *, CTD-2374C24.1, SYN3*	*RP11-6N13.1* *, SBF1, USH2A, SEMA6D*
Nose morphology	*—*	*HS3ST4*	*—*	*AC004158.2, VPS13B*
Nose width	*DSCAML1, EIF2B5*	*—*	*PCCA*	*IKZF2*
Nose size	*DLC1, EIF2B5*	ROBO1*, LINC00886, BMP7, RAD51B, CACNA2D3	*MSRA*	ROBO1*, *MSRA*

Note.—Adaptive genes shared by at least two of the four populations are highlighted: adaptations shared among populations across the Wallace–Huxley’s line are marked with asterisks; those presented in only one side of the Wallace–Huxley’s line are underlined. Trait-associated genes are reported by the GWAS catalog. ADM, Andaman Negrito; MLS, Malaysian Negrito; PHI, Philippine Negrito; PNG, Papuan.

### Evolution Scenarios of Pigmentation-related genes in the TIAs

To characterize the genetic architecture of skin pigmentation primarily in the four representative TIA populations (again, some populations with enriched bASN ancestry and even darker skin pigmentation, such as Melanesians from Bougainville, were excluded due to limited samples size), we compiled a comprehensive list of 1,057 pigmentation genes in human from previous genetic studies or biological pathways (supplementary table S3, Supplementary Material online, see Materials and Methods). At 103 of these variants that affect 85 genes (supplementary table S4 and figs. S13–S15, Supplementary Material online), the risk alleles were reported in previous association studies and were further determined as dark pigmentation alleles (DPAs) reported in the Africans or light pigmentation alleles (LPAs) reported in the Eurasians (see Materials and Methods). Since most of these risk alleles were identified under the additive model in association studies, we assumed that the two alleles at a candidate SNP have opposite effects on human coloration. Approximately 88.8% of the pigmentary variants are standing variants in the Africans, and 11.2% or less (some DPAs possibly drifted out in the Africans after the “Out of Africa” event) of them are possible de novo DPAs. The pigmentary variants collected in this study were biased to the LPAs reported in Europeans because previous association studies were conducted predominantly in individuals of European descent. Moreover, the LPAs reported in Europeans and those reported in East Asians (although with a limited number) were specifically enriched in these two populations, respectively (supplementary fig. S16*A*, Supplementary Material online), supporting the convergent evolution of the light skin phenotype in Europeans and East Asians. When focusing on the DPAs reported in Africans, we revealed a substantial excess of DPAs in the Africans and the representative TIA populations over those in Europeans and East Asians (supplementary fig. S16*B* and *C*, Supplementary Material online), thereby confirming the genetic basis for the dark skin phenotype.

**Fig. 3.**
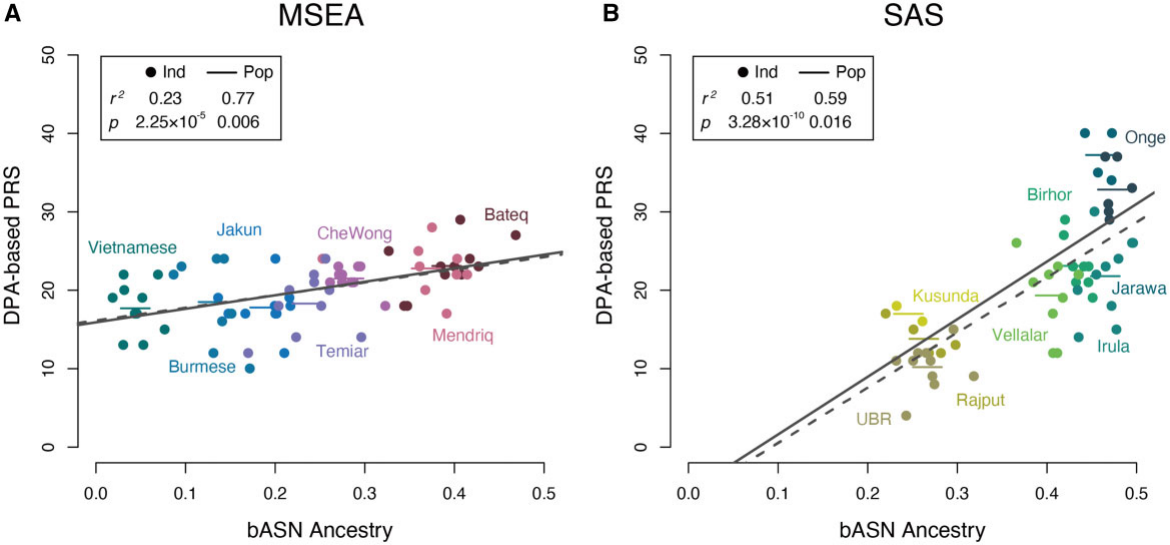
Polygenic risk score calculated based on 24 dark pigmentation alleles (DPAs) reported in the Africans are positively correlated with the bASN ancestry. Each dot represents an individual, and the short horizontal line indicates the mean level across samples in a population. The gray-dashed lines are linear regressions on individuals, and the gray solid lines are those on populations. MSEA, mainland Southeast Asia; SAS, South Asia.

**Fig. 4.**
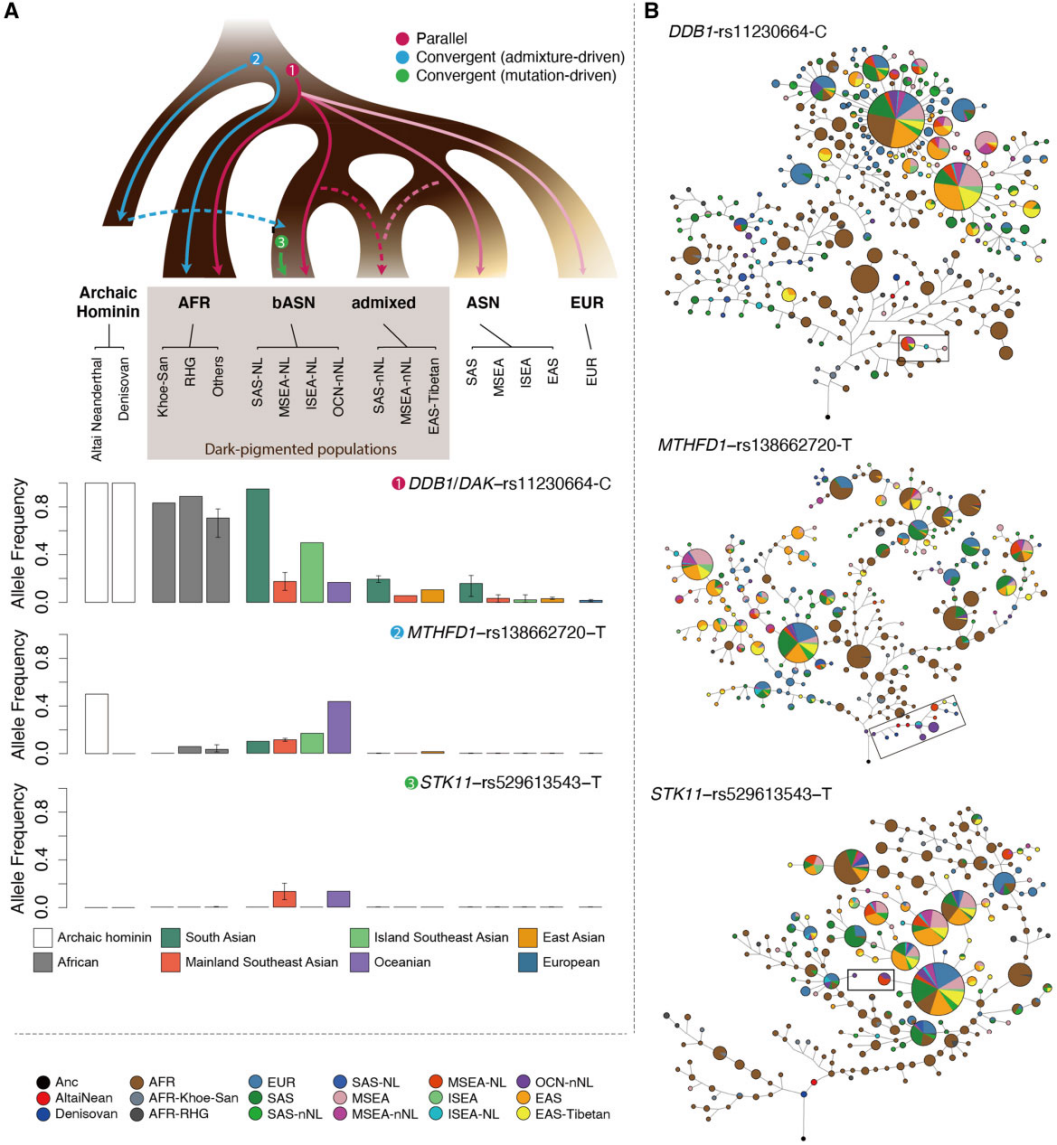
A putative model of pigmentary variants evolution. (*A*) A simplified model involving three possible scenarios of pigmentary variants evolution. The evolutionary path and frequency distribution for the dark pigmentation alleles (DPAs) of several outstanding examples are shown in the model. In the four examples, *DDB1*/*DAK*−rs11230664−C is associated with skin pigmentation in Africans, and the other two are located in the enhancer/promoter regions of genes involved in the response to UV radiation. (*B*) Haplotype networks for each example in (A). Haplotypes carrying the key DPAs are framed in black-edged boxes. RHG, rainforest hunter-gatherer; NL, Negrito-like; nNL, non-Negrito-like.

We hypothesized that the dark pigmentation of the TIAs may have originated from the bASN ancestry, as we observed that the DPA-based unweighted polygenic risk score (PRS)—number of DPAs in each individual—is positively correlated with the bASN ancestry in mainland Southeast Asia (*r*^2^=0.23 and *P* = 2.25 × 10^−5^ across individuals; *r*^2^=0.77 and *P* = 0.006 across populations) and South Asia (*r*^2^=0.51 and *P* = 3.28 × 10^−10^ across individuals; *r*^2^=0.59 and *P* = 0.016 across populations) (fig. 3 and supplementary fig. S17, Supplementary Material online). We further analyzed the flanking haplotypes of the key DPAs reported in Africans, and found that most of these are IBD in the African and non-African populations and are likely to be the ancestral states of the modern human, implying possible parallel evolution. Notably, the derived allele (G) at rs872257, an intronic variant of the antisense gene *RP11-125B21.2*, had an excess of frequency in all the African populations, NL-TIAs, and PNG (allele frequency [AF]=0.5–0.94) over the other Eurasians (AF=0.20–0.45), and two DPAs (rs6510760-A and rs112332856-C) in *MFSD12* were enriched in the TIA populations in each geographic region. However, in most of the cases, the DPAs were lost in several of the TIA populations during evolution. For instance, we observed the accumulation of three *DDB1*-*DAK* DPAs (rs11230664-C, rs376397-G, and rs2513329-T) in ADM and PHI (0.95 in ADM; 0.5 in PHI; 0.17–0.89 in the Africans; 0–0.23 in other Eurasians), which are in strong linkage disequilibrium (LD) with the aforementioned missense mutation at rs2260655 (fig. 4). Although selective sweep at this site was only detected in PHI, we suspect that these DPAs are also beneficial in ADM as these show extremely high frequency and share homologous haplotypes with those in PHI. Other examples include two variants (rs6497271-A and rs4932620-T) in *HERC2* in ADM (AF=0.5 in ADM; AF<0.23 in the nonindigenous Eurasians). In the DPAs reported in Europeans, we also found such examples like the ancestral allele (T) at an intronic variant *TRPS1*-rs2737212 (AF>0.9 in the Africans and the representative TIAs; AF=0.52–0.89 in the nonindigenous Eurasians). As for other pigmentary variants without determined effect alleles associated with pigmentation or depigmentation, we assumed that the alleles showing higher frequencies in the representative TIAs than in the nonindigenous Eurasians are more likely to be DPAs (hereafter denoted as putative DPAs). Interestingly, the population-specific putative DPAs, presenting in at least one representative TIA population but missing in all the nonindigenous Eurasians (see Materials and Methods), tend to show in the Africans (*P* = 1.7 × 10^−6^, odds ratio (OR)=0.36, Fisher’s exact test), whereas those population-specific non-pigmentary alleles are more likely to be novel mutations in the representative TIAs (*P* = 3.2 × 10^−12^, OR=1.5, Fisher’s exact test) (supplementary table S5, Supplementary Material online). For example, we found a 112.6-kb haplotype involving two PNG-specific missense pigmentary alleles (rs7699637 and rs7682296; AF=0.1 in PNG) in *FRAS1*. This haplotype is constituted of 60 alleles, including 42 putative DPAs, and is likely an ancient haplotype harbored by the archaic hominins and some African individuals. A similar example is a 21.5-kb haplotype carrying a highly conserved regulatory variant (rs113432620-G; an MLS-specific allele, AF=0.25) and other 13 putative DPAs at *RP11-6N13.1*. The allele age at rs113432620-G was estimated to be older than 400 ka. This result, again, suggests that dark skin in the TIAs could be one of the most important phenotypes inherited from African ancestry. However, the adaptation of inherited DPAs mingled with that of LPAs in the skin-color determination of TIAs. We identified adaptation at the *OCA2* loci in the TIA populations and found that the adaptive alleles matched the Asian prevalent alleles, which could be a consequence of recent admixture (supplementary fig. S16*D*, Supplementary Material online).

**Fig. 5.**
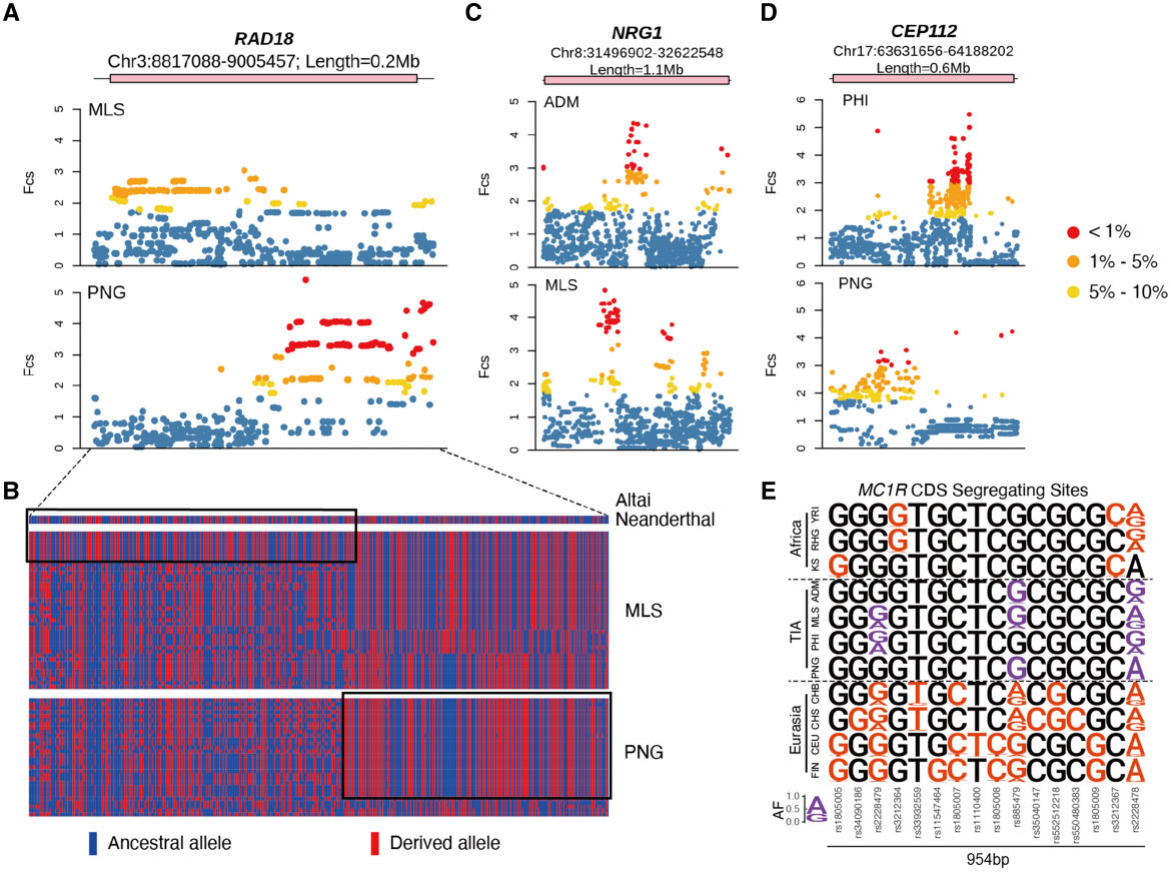
Examples of convergent evolution on the gene level. (*A*) Differentiated signals of adaptation at *RAD18* in the Malaysian Negritos (MLS) and the Papuans (PNG). (*B*) Haplotypes structure of *RAD18* in the Altai Neanderthal, MLS, and PNG. Each row indicates a haplotype. Only segregating sites with minor allele count >2 in the total sequences are shown in this plot. The adaptive haplotype in MLS is likely an introgressed haplotype from the Altai Neanderthal (denoted by the top-left black-edged box), whereas that of PNG is inherited from the modern human ancestry (denoted by the bottom-right black-edged box). (*C*) Distinct adaptive signals in Andaman Negritos (ADM) and MLS at *NRG1*. (*D*) Distinct adaptive signals in the Philippine Negritos (PHI) and PNG at *CEP112*. In (*A*), (*C*), and (*D*), the pink bar indicates gene location. (*E*) Variation in the coding sequence (CDS) of *MC1R*. Segregating sites in each population are shown in color. AF is proportional to the height of the letter.

In addition, gene flow plays an essential role in facilitating the convergent evolution of dark pigmentation. For instance, the only putative DPA shared by the four representative TIA populations, *MTHFD1*-rs138662720-T, is related to different haplotypes between Africans and TIAs. The haplotypes in TIAs are similar to the Altai Neanderthal sequences and were inferred to be introgressed haplotypes by *ArchaicSeeker 2.0* (Yuan et al. 2021). They were clustered on an independent basal branch in the haplotype network, whereas those in Africans were on the shared branches with other Eurasians (fig. 4). *MTHFD1* plays a role in the folate-mediated 1-carbon transfer process, which is influenced by the environmental UV levels and possibly evolves in parallel with skin pigmentation (Jones et al. 2018). Interestingly, rs138662720 is located in the promoter and enhancer elements of *MTHFD1* with signals of H3K4me3+ and H3K27Ac+ in melanocyte, and thus may affect *MTHFD1* expression (supplementary fig. S18, Supplementary Material online). Archaic introgression also facilitates the convergent evolution at different sites of the same gene across the TIA populations. One notable example is *RAD18*, which is recruited to replication forks and facilitates the DNA repair after UV exposure (Liu et al. 2012). MLS and PNG showed differentiated signals of positive selection at this gene, and the adaptive haplotype in MLS is likely an introgressed haplotype from Altai Neanderthal (fig. 5*A* and *B*). This haplotype reaches a frequency of 0.23 in MLS, but is absent in other worldwide populations. We also observed that some TIA populations share new mutations in the pigmentation-related genes. The derived allele (T) at rs529613543 in *STK11* (also known as *LKB1*), a protein-coding gene modeled as an UV-induced DNA damage sensor in mice (Esteve-Puig et al. 2014), specifically presented in MLS (AF=0.14) and PNG (AF=0.13) (fig. 4). The allele age was estimated to be 5.0 and 8.8 ka in the two populations, respectively. Although identical mutations across human populations more likely reflect an ancestral event, we could not rule out the possibility of independent de novo mutations in this case. This locus is around the enhancer and promoter region of *STK11* with high DNase sensitivity (supplementary fig. S19, Supplementary Material online). Therefore, we speculated any mutations at this locus could have altered the pattern of gene expression thence phenotypic consequences.

Hard sweeps of population-specific de novo mutations may also contribute to skin color evolution in TIA populations. For instance, a 36.9-kb haplotype in *USH2A* was under strong positive selection in PNG (AF=0.43) (supplementary fig. S20*A*, Supplementary Material online). The adaptive haplotype could have been introduced to PHI (AF=0.17) via gene flow. A recent study showed that *USH2A* showed abnormal pigment deposition and reduced expressions of some melanin metabolism-related genes, such as *TYR* and *OCA2*, in retinal cells derived from induced pluripotent stem cells, indicating potential involvement of *USH2A* in the pigmentation pathway (Deng et al. 2017). Moreover, we identified some de novo mutations with severe consequences and under adaptation, and thus could have functional importance, such as rs201616507, a missense mutant carried by a 436.2-kb extended haplotype in *ZRANB3* in PHI (AF=0.22) (supplementary fig. S20*B*, Supplementary Material online). *ZRANB3* functions in response to UV exposure by regulating DNA replication and repair (Vujanovic et al. 2017).

### Genetic Convergence of Pigmentation on the Gene Level

Although identical adaptive variants were identified across the TIA populations, considerable variations showed how different a pigmentation gene functions in response to the selective pressure on skin color in different populations. Except for the aforementioned example of *RAD18*, several examples of convergent evolution at the gene level were discovered. Notably, *NRG1* involved in melanocyte development (Buac et al. 2009) showed distinct signals in ADM and MLS (fig. 5*C*); different *CEP112* signal loci were identified in PHI and PNG (fig. 5*D*), and this gene is associated with iris color in South Asians (Jonnalagadda et al. 2019).

Previous studies have suggested that the dark skin phenotype in Africans could be a result of strong functional constraints on pigmentary genes, whereas relaxation of the constraints in the Eurasians leads to the light skin phenotype in these populations. *MC1R* is an exemplar gene under purifying selection in the Africans (Harding et al. 2000; Sturm et al. 2001; Makova and Norton 2005). In our data, we found a significant excess of mutation burden on the collective pigmentation-related genes compared with the whole genome in most Eurasian populations, except for some TIAs such as PNG and ADM (supplementary fig. S21*A*, Supplementary Material online). This observation may be largely attributed to the accumulation of functional mutations in the Eurasian populations, as most of the pigmentation-related genes were reported in these populations, and to a lesser extent, constraint relaxation could have occurred in some genes in Eurasians. *MC1R* showed signs of purifying selection in PNG and ADM, including the low mutation burden and small nucleotide diversity in the coding sequences of this gene that are comparable to those in Africans (supplementary fig. S21*B* and *C*, Supplementary Material online). PNG and ADM also showed a close genetic affinity with Africans on this gene (supplementary fig. S21*D*, Supplementary Material online). Moreover, the segregating sites in the four representative TIA populations were highly consistent (fig. 5*E*). These polymorphisms were also observed in the other Eurasian populations but did not overlap with those shown in Africans. These results suggest that purifying selection of *MC1R* could be a consequence of convergent evolution in the African and Asian indigenous populations.

## Discussion

In this study, we reveal that different geographical TIA populations are genetically distinct from each other, but also provide compelling evidence for the shared ancient Asian ancestry of TIAs. Our analysis showed no supporting evidence that the identified bASN ancestry resulted from shared evolution of archaic introgression, recent admixture, or other random effects. Rather, it is more likely a sign of an independent pulse of human migration to Asia, involving the ancestors of the present-day Negritos and Oceanic populations. Previous genetic studies focusing on ancient genomes or single present-day indigenous populations have suggested an ancient initial occupation of Southeast Asia, followed by several layers of migrations from different ancestry sources and different places (Rasmussen et al. 2011; Xu et al. 2012; Lipson et al. 2018; McColl et al. 2018). The whole-genome sequencing data of diverse TIA populations we analyzed poses an additional advantage on tracing the ancestry footprints in the present-day human, and connecting the genomes with traits.

Taking advantage of the whole-genome sequences that we generated for the Malaysian Orang Asli samples and those collected from other published studies, we present the first genome-wide survey of local adaptations in multiple geographically dispersed groups of TIA populations and show that these populations have adapted to the challenging habitats of the tropical rainforest via both shared and distinct genetic determinants. For instance, we found evidence of strong sweeps shared by the four representative TIA groups, targeting a hair-associated gene *LIMS1*, and a height-related gene *COL24A1*. Evidence for the shared adaptations of the Negrito-like phenotypes across TIA populations was elaborated on the gene level and the pathway level. Meanwhile, plenty of differentiated adaptations were reported by previous studies and also observed in our data.

An inevitable issue regarding the studies on TIA populations is the genetic mechanism of the dark skin color phenotype. We explored the dark pigmentation evolution primarily based on the four representative TIA populations for several advantages, including outstanding dark skin phenotype evidenced by the literature, long population history supported by the enriched bASN ancestry, and less genetic interaction with the surrounding nonindigenous populations compared with other nNL-TIAs. This study provides a full landscape of the pigmentary variants and genes in TIA populations. Despite the lack of a comprehensive assessment of the skin color phenotype in the samples studied, we referred to the reported data in previous studies, and found that the TIA populations we focused on here have a higher melanin index than other populations inhabiting similar latitudes (supplementary note S1, Supplementary Material online). Therefore, it is conceivable to summarize the overall pattern of skin color evolution based on the genetic architecture of pigmentary variants in populations qualitatively differentiated in skin color. The priori pigmentary variants were obtained from the GWAS studies primarily carried out in the major continental groups, as very few results were reported in other minority populations. Kenny et al. (2012) reported a missense variant in *TYRP1* that is associated with blond hair in Melanesians, but this variant was not captured in the sequences we analyzed.

Five conclusions can be drawn from the present analyses. First, the dark skin phenotype is likely characterized by extreme genetic heterogeneity, as a higher genetic load has been observed in the indigenous populations than in the nonindigenous populations in each geographic area. Second, a large number of the DPAs are likely to be IBD among TIA populations and were probably inherited from the modern human ancestry. These results support that the early dispersal of the dark-skinned people out of Africa was not accompanied by significant changes in skin color (Jablonski 2018), and this is consistent with an individual case observed by Crawford et al. (2017). Third, we observed some putative DPAs are likely de novo mutations in TIAs, or adapted from the archaic introgression, suggesting the possibility of convergent evolution. Previous studies have suggested convergent evolution of light skin in Europeans and East Asians and that of the dark skin among African groups (Norton et al. 2007; Martin et al. 2017), and our analyses complement the hypothesis by providing additional supporting evidence. Fourth, the convergent purifying selection also drives pigmentation in the tropical indigenous populations, such as *MC1R* in the Africans, PNG, and ADM. A previous study did not support the strong selective constraint on *MC1R* in the Melanesians (Norton et al. 2015), possibly due to the genetic differentiation between near and remote Oceanic populations. Finally, some of the LPAs are also adaptive in TIA groups, which could evolve from standing variants or gene flows between populations, again suggesting the complexity of the skin color phenotype. Based on our data, we conclude that the phenotypic convergence in skin color across the geographically diverse TIA populations is likely a joint consequence of shared genetic ancestry and convergent molecular adaptation.

Our results supported the hypothesis that the dark-pigmented skin evolved in early hominins in Africa, and that formed the substrate of the skin color of the ancient Asian ancestry. Populations dispersing and living along the coasts of southern and southeastern Asia in the Late Pleistocene experienced strong and seasonally relatively invariant UV levels, and that favored the maintenance of dark pigmentation, probably involving selection on ancestral gene variants and recent mutations (Jablonski 2021). Evidenced by the adaptive introgressed haplotypes in *MTHFD1* and *RAD18*, we established the genetic contribution of Neanderthals to the skin pigmentation in TIA populations, which was not recognized before despite that such connection was only identified in the light-skinned modern Eurasians (Dannemann and Kelso 2017; Rotival and Quintana-Murci 2020). Human skin pigmentation is jointly determined by different combinations of genetic and cultural conditions over time and through space (Jablonski 2021). Our results showed that UV-induced folate degradation could be related to the dark skin phenotype in TIA populations. *MTHFD1* plays a role in the development of neural tube defects—a congenital disorder due to folic acid deficiency in pregnant mothers. Therefore, adaptation at *MTHFD1* may help to improve reproductive success. Notably, folate-dependent reproduction is not necessarily a driving force of human skin color adaptation to UV. *MTHFD1* and other folate-related genes could have evolved in parallel with the causal variants determining pigmentation (Jones et al. 2018). Further studies are needed to investigate the interaction of folate, skin pigmentation, and UV conditions, and to figure out how the identified *MTHFD1* variant affects skin pigmentation. In addition to UV, selective pressures on the immune function could also be a reason, as eumelanin is important in enhancing the barrier function of the skin. The tropical indigenous populations have been facing extreme epidermal environments, and some of the adaptive genes (e.g., *LRBA*) we identified are involved in immune processes. It should be noted that the effects of material culture and traditions must also be considered, even if the strength of these influences cannot be easily quantified. Some TIA populations usually wear few clothes and their diets were rich in fish (Bolton 1972; Sreenathan et al. 2008; Headland 2015), and these may have contributed to the selective constraint on pigmentation. It was also proved that dark-pigmented people can achieve sufficient vitamin D with adequate duration of sun exposure (Nimitphong and Holick 2013).

Despite the lack of experimental evidence for direct connection between the shared adaptive alleles and the shared phenotypes across TIA populations, those specific patterns of population allele sharing and reported genotype–phenotype associations could help to establish such connections. We caution that the model provided in the present study is greatly simplified. Since human skin color is a highly complex polygenic trait, we would not be able to quantify the effects of parallel and convergent adaptation without variant functional validations. However, our efforts on demonstrating the molecular evolutionary pattern enable the life science communities to better understand the human evolutionary process via skin color adaptation, and provide insights into the nature of parallel or convergent evolution. With in-depth genetic and phenotypic studies, as well as functional assessments of the putatively selected variants, it is therefore plausible to characterize genetic mechanisms of human skin color adaptation.

## Materials and Methods

### Sample Collection and Data Processing

Peripheral blood samples were collected from 50 Orang Asli Individuals from Peninsular Malaysia (supplementary note S2, Supplementary Material online). Written informed consent was obtained from all participants. Research/ethics approval and permits were obtained from the Research and Ethics Committee of Universiti Teknologi MARA [Ref no: 600-RMI (5/1/6)], the Department of Orang Asli Development (Jabatan Kemajuan Orang Asli Malaysia, JAKOA) [JHEOA.PP.30.052.Jld 5(17)], as well as the district offices, village chief, and the chairperson of the Committee of Village Development and Security. Sample collection was also approved by the Biomedical Research Ethics Committee of Shanghai Institutes for Biological Sciences (ER-SIBS-261903). The family history, pedigree, and self-reported ethnicity were recorded via an interview using the local dialect. All procedures were in accordance with the ethical standards of the Responsible Committee on Human Experimentation (approved by the Biomedical Research Ethics Committee of Shanghai Institutes for Biological Sciences) and the Helsinki Declaration of 1975, as revised in 2000.

All the samples were sequenced at 30× coverage for 150-bp paired-end reads, using Illumina Hiseq X10 version 2.5 by Wuxi NextCODE in Shanghai. The raw reads were aligned to the reference GRCh37 human genome using BWA (Li and Durbin 2010). The low mapping quality reads (MAPQ<20) and potential duplicates were removed using SAMtools (Li et al. 2009) and Picard toolkit, respectively. We then realigned the indels using IndelRealigner from GATK (McKenna et al. 2010), and used the GATK variant quality score recalibration to adjust the base quality score bias from the sequencer. Variant calling was performed using HaplotypeCaller (McKenna et al. 2010; DePristo et al. 2011). After filtering out the low-quality variants, we finally obtained 51,722,790 biallelic SNPs for further analyses. All of these samples were self-reported to be unrelated, but we identified first-, second-, and third-degree relatedness involving 21 of them, as suggested by KING version 2.1.2 (Manichaikul et al. 2010). Considering that these indigenous populations are highly inbred, we only removed four individuals showing the first-degree relatedness and combined the rest genomes with public data sets. A detailed description of samples and data can be found in supplementary note S2, Supplementary Material online.

### Population Data Collected from Public Resources

We obtained the following data sets of present-day modern humans to contextualize the TIA samples (supplementary note S2, Supplementary Material online): 1) the 1000 Genomes Project Phase 3 data (1000 Genomes Project Consortium 2015); 2) the Simons Genome Diversity Project (Mallick et al. 2016); 3) the Estonian Biocentre Human Genome Diversity Panel (Pagani et al. 2016); 4) high-coverage genomes of indigenous populations from South and East Asia (Lu et al. 2016, 2017; Mondal et al. 2016); 5) high-density SNP-array data of worldwide populations (Reich et al. 2011; Henn et al. 2012; Schlebusch et al. 2012; Petersen et al. 2013; Pugach et al. 2013; Deng et al. 2014; Ko et al. 2014; Lazaridis et al. 2014; Patin et al. 2014; Aghakhanian et al. 2015; Liu et al. 2015). We generated two combined data sets. One was compiled from all the sequencing and SNP-array data, including 49,545 autosomal SNPs of 6,059 samples, which was used for inferring the general genetic relationship and ancestry makeup of populations; The second was restricted to the genome sequences, including 17,242,884 autosomal SNPs of 2,845 samples, used for the investigation of genome variations. Archaic and ancient data used in this study include: 1) high-coverage genomes of the archaic hominins, including Altai Neanderthal (Prüfer et al. 2014) and Denisovan (Reich et al. 2010); 2) worldwide Neolithic-to-Paleolithic ancient human genomes (Allen Ancient DNA Resource, https://reich.hms.harvard.edu/allen-ancient-dna-resource-aadr-downloadable-genotypes-present-day-and-ancient-dna-data, last accessed December 29, 2021, version 44.3).

### Variants Annotation, Genotype Imputation, and Phasing

Functional consequences and the pathogenicity of the variants were assessed using the Variants Effect Predictor (McLaren et al. 2016). The ancestral allele at each variant was determined according to the ancestral sequences (e71) released by the 1000 Genomes Project. Variants were mapped to genes according to Ensembl 96 (GRCh37). Functional enrichment for genes was obtained using the annotation tool clusterProfiler v3.10.1 (Yu et al. 2012) and ToppGene (https://toppgene.cchmc.org/enrichment.jsp, last accessed December 29, 2021) (Chen et al. 2009). Haplotype phasing and imputation were carried out using Shapeit2 (Delaneau et al. 2011) (supplementary note S2, Supplementary Material online).

### Collection of the Pigmentation-Related Pathways, Genes, and Variants

We focused on the evolution patterns of some genes of particular interest, including genes involved in pigmentation-related pathways and ontologies defined by KEGG and Gene Ontology (provided by clusterProfiler v3.10.1; Yu et al. 2012), and genes previously reported to be associated with pigmentation (including iris, hair, and skin color with a shared genetic basis to some extent; Rees 2003; Sulem et al. 2007) in human populations (supplementary table S3, Supplementary Material online). The final priori pigmentary gene list contains 1,057 genes in total. A list of 103 pigmentary alleles was further selected from the priori pigmentary variants for their reported effects on pigmentation or depigmentation in previous association studies (supplementary table S4, Supplementary Material online). According to the relative effects of the alleles shown in the additive model of association studies, we named the two alleles at a site as LPA and DPA, respectively.

### PCA and Inference of the Ancestry Components

We carried out approximate LD-pruning of SNPs by selecting a SNP in each of the 200-kb nonoverlapping windows, and then performed flashPCA (Abraham and Inouye 2014) and ADMIXTURE version 1.3.0 (Alexander et al. 2009) with all the options set to default. We specified *K* = 2–26 in the ADMIXTURE analysis and performed ten replications for each *K* using random seeds. The cross-validation error was measured to assess the number of clusters with the best fit for each analysis.

### Population Genetic Differentiation (*F*_ST_)

*F*_ST_ was assessed following Weir and Cockerham (1984) to evaluate the overall population genetic distance and to identify local regions that are highly differentiated among the representative TIA populations. *F*_ST_ was calculated for every single SNP, and was then weighted over the whole genome to construct the population phylogeny shown in figure 1*A*. We also estimated the four-population *F*_ST_ for the four representative TIA populations, based on a 10-kb sliding window along the chromosome, stepping by 1 kb. The four-population *F*_ST_ value for each gene was represented by that of the top window overlapping this gene. We normalized the *F*_ST_ of all genes by square roots transformations, and genes showing normalized *F*_ST_>1.96 were selected as outliers.

### Mitochondrial DNA and Y-Chromosome Analysis

The mtDNA haplogroups were identified using HaploGrep2 (Weissensteiner et al. 2016) based on PhyloTree 17 (van Oven 2015). The Y chromosome haplogroups were determined based on known Y-SNP markers from the International Society of Genetic Genealogy (ISOGG) phylogenetic tree 2019–2020 using in-house scripts. The Filippino mtDNA haplogroups were obtained from 75 individuals from Delfin et al. (2014). We filtered out individuals with a lower resolution of haplogroup classification, including the individuals with an overall rank <0.6 (provided by HaploGrep2; Weissensteiner et al. 2016) for the mtDNA, and those with estimated Y haplogroups staying on the ISOGG Y-DNA phylogenetic tree trunk. We performed BEAST v.2.6.0 (Bouckaert et al. 2014) to estimate the coalescent time of haplogroups. The age of mtDNA haplogroup MN (77,000 years, 95% CI=61,400–93,200) (Fu et al. 2013) and NRY haplogroup CT-M168 (71,760 years, 95% CI=69,777–73,799) (Karmin et al. 2015) were used for calibration of age estimation of mtDNA and NRY haplogroups, respectively.

### Identification of the bASN-Derived Alleles

We inferred the bASN-derived alleles from the whole-genome sequences of ADM, MLS, PHI, and PNG, following Watanabe and Ohashi (2020). We first identified alleles with frequency>0.2 in any of the above four populations but <0.1 in European and East Asian populations. Considering possible gene flow between TIA and the surrounding non-TIA populations in South and Southeast Asia, we did not exclude those alleles carried by South and Southeast Asians. Next, LD coefficients (*r*^2^) were calculated between SNPs located within 500 kb from each other using PLINK v1.90 (Purcell et al. 2007). Alleles with *r*^2^>0.5 were counted in as bASN-derived alleles.

### Estimating the GS of bASN-Derived Alleles

We estimated the unweighted GS of the bASN-derived alleles in each ancient sample as follows:

$${\mathrm{GS}}_{\mathrm{unweighted}}=\frac{1}{m}\sum_{i=1}^{m}d_{i},$$

where *m* is the number of bASN-affected SNPs genotyped in the individuals and *d_i_* is the presence of the bASN-derived alleles at SNP. The 14,353 bASN-derived alleles were assigned into 1,551 blocks using a threshold of between-SNP-distance of 500 kb. We then calculated the weighted GS for each sample as:

$${\mathrm{GS}}_{\mathrm{weighted}}=\frac{1}{N}\sum_{i=1}^{N}\frac{i}{n_{j}}\sum_{i=1}^{n_{j}}d_{ij},$$

where *N* is the number of blocks, *n_j_* is the number of bASN-derived SNPs in the block *j*, *d_ij_* is the presence of the bASN-derived alleles at SNP *i* of block *j*. We used linear regression to examine the association between the GS and time for samples. Samples with a total number of genotyped SNPs less than the first quantile of all the ancient samples were excluded from this analysis. Geographic coordinates of the samples, that is, latitude and longitude, and the first ten PCs were included as covariates in the model:

$$\mathrm{GS}=\alpha +\beta_{\mathrm{time}}X_{\mathrm{time}}+\beta_{\mathrm{lat}}X_{\mathrm{lat}}+\beta_{\mathrm{lon}}X_{\mathrm{lon}}+\beta_{\mathrm{PC}1}X_{\mathrm{PC}1}+\beta_{\mathrm{PC}2}X_{\mathrm{PC}2}+\cdots +\beta_{\mathrm{PC}10}X_{\mathrm{PC}10}.$$

### Modeling of Admixture Graphs

We first applied AdmixtureBayes (Nielsen 2018) to estimate the top posterior admixture graphs, based on the covariance matrix of the AF profiles. Only 148,416 variants with AF in 0.05–0.95 in any of the populations used in our study and those with between-SNP-distance >10 kb were used in this analysis. For each AdmixtureBayes run, a total of 1,000,000 MCMC steps were carried out, with other parameters set to default. Multiple sets of populations were analyzed to obtain a full picture of the admixture graphs, with each graph inferred from an independent data set. The chimpanzee was used as the outgroup. We finally input the graphs estimated from AdmixtureBayes to qpGraph (Patterson et al. 2012) to test the goodness of fit. We accept only the graphs with an absolute value of the *Z*-score of the worst *f*_4_-statistic <3. The parameters of qpGraph include: outpop=NULL, blgsize=0.05, lsqmode=YES, and diag=0.0001.

### Constructing the Haplotype Networks

Given the frequency (*f*) of an allele of interest, we identified the related haplotypes for this allele using the following procedures. First, we screened for the AF block involving this allele in each by 1) screening for the alleles at frequency *f* in the nonoverlapping 50-kb windows, 2) selecting out the windows with at least five alleles at frequency *f*, and 3) merging the adjacent windows and selecting the one with the target variant included. Second, we collected the union of the AF blocks identified in each population. Then, the haplotype networks were constructed and visualized at genomic regions of interest using NETWORK 10.1.0.0. To construct the haplotype networks, we selected 50% of the samples in populations with sample size >50, and make sure that the samples carrying the target alleles are included. We first calculated the median-joining network (Bandelt et al. 1999), and then removed the superfluous nodes using the maximum-parsimony algorithm (Polzin and Daneshmand 2003).

### Detection of Natural Selection

We calculated iHS using *Selscan v1.2.0* (Szpiech and Hernandez 2014) for polymorphic sites in each population, and LSBL for whole-genome variants using in-house scripts. For each SNP, we combined iHS and LSBL scores into a Fisher’s score (*F*_CS_) following Lopez et al. (2019):

$$F_{\mathrm{cs}}={-\log}_{10}\left(p_{\mathrm{iHS}}\right)+{-\log}_{10}\left(p_{\mathrm{LSBL}}\right),$$

where *p* is the empirical *p* value across the genome. When calculating the LSBL statistic, we chose Han Chinese (CHB) as an outgroup, and a neighbor population with the closest genetic relationship (measured by *F*_ST_) as a reference for each representative TIA population: BEB for ADM, Burmese for MLS, and Dusun for PHI and PNG. An outlier SNP was defined as an SNP with an *F*_CS_ among the 5% highest of the genome. A putatively selected genomic region was defined as a 100-kb window presenting a proportion of outlier SNPs among the 5% highest of all windows, in four bins of SNP numbers. Windows containing less than ten SNPs were discarded.

### Estimating the Allele Age

Allele ages were estimated using the Genealogical Estimation of Variant Age (GEVA) (v1 beta) based on the mutation clock model (Albers and McVean 2020). The initial effective population size and mutation rate were set to be 10^4^ and 1.25 × 10^−8^, respectively, as recommended in the manual.

### Estimating Mutation Load

Variants were grouped into three categories according to the genomic evolutionary rate profiling (GERP) score, corresponding to different deleterious levels (2–4, mild; 4–6, moderate; >6, critical). We assigned a selection coefficient (*s*) for variants in each category following Boyko et al. (2008): *s*=−4.5 × 10^−4^ for GERP in 2–4, *s*=−4.5 × 10^−3^ for GERP in 4–6, and *s*=−1 × 10^−2^ for GERP >6. The total mutation load (*L*) is the sum of load for each locus (Kimura et al. 1963) under an additive model:

$$L=2shf\left(1-f\right)+sf^{2}$$

where *f* is the derived AF, and *h* = 0.5. We estimated *L* for the whole genome, for a set of genes (e.g., pigmentary genes), and the coding sequence region of *MC1R* in this study. To make it comparable across genes or gene sets, we averaged *L* by SNPs.

## Declarations

### Ethics Approval and Consent to Participate

Written informed consent was obtained from all the Malaysian Orang Asli participants. Research/ethics approval and permits were obtained from the Research and Ethics Committee of Universiti Teknologi MARA [Ref no: 600-RMI (5/1/6)], the Department of Orang Asli Development (Jabatan Kemajuan Orang Asli Malaysia, JAKOA) [JHEOA.PP.30.052.Jld 5(17)], as well as the district offices, village chief, and the chairperson of the Committee of Village Development and Security. Sample collection was also approved by the Biomedical Research Ethics Committee of Shanghai Institutes for Biological Sciences (ER-SIBS-261903). The family history, pedigree, and self-reported ethnicity were recorded via an interview using the local dialect. All procedures were in accordance with the ethical standards of the Responsible Committee on Human Experimentation (approved by the Biomedical Research Ethics Committee of Shanghai Institutes for Biological Sciences) and the Helsinki Declaration of 1975, as revised in 2000.

### Consent for Publication

Not applicable.

## Supplementary Material

Supplementary data are available at *Molecular Biology and Evolution* online.

## Supplementary Material

msab361_Supplementary_Data
