# Supplementary material for: Genetic Connections and Convergent Evolution of Tropical Indigenous Peoples in Asia
Source: Mol Biol Evol. 2021 Dec 23;39(2):msab361. doi: 10.1093/molbev/msab361 (PMC8826522; doi:10.1093/molbev/msab361)
Supplement: msab361_Supplementary_Data [file msab361_supplementary_data.zip › MBE-21-0591-final_supplementary_materials.pdf]

Supplementary Materials

Supplementary note S1. Overview of skin pigmentation of the tropical indigenous Asian (TIA) populations. Dark skin color is strongly associated with populations living in the equatorial region or at highlands where the ultraviolet (UV) radiation is much more intensive and longer-lasting than other places of the world (supplementary fig. S1.1, S1.2). It is especially enriched in the indigenous populations, such as the hunter-gatherers in the tropical forests. Critical examination of human skin color variation strongly supports that skin color is an adaptive trait (Chaplin and Jablonski 1998; Jablonski and Chaplin 2000; Jablonski and Chaplin 2010). Dark skin may help to confer fitness benefits in response to the overexposure of UV, such as preventing sunburn damage that could result in skin cancers, and preserving the body's supply of folate (Armstrong and Kricker 2001; de Gruijl, et al. 2001; Brenner and Hearing 2008; He, et al. 2009; Jablonski and Chaplin 2010; Juzeniene, et al. 2010). Cultural factors like diet, activity schedules, use of clothing and shelter, as well as mate selection might also have some effects on the evolution of skin color in some populations (Jablonski 2004; Štěrbová, et al. 2019).

Melanin index has been measured for only several TIA populations in previous studies, and related data are summarized in supplementary table S1.1. Apparently, the Malaysian Negritos and Papuans show higher melanin index than most of the global populations, except those with African ancestry. Although we could not find data of Philippine Negritos and Andamanese, their dark skin color has been widely described in numerous anthropological studies. For instance, David Barrows described the body color of Philippine Negritos as "dark brown, several shades darker than the Malay" (Barrows 1910); Gopal Krishan reported the nuances of the skin color of Onge as "reddish-brown to black" (Krishan 1993).

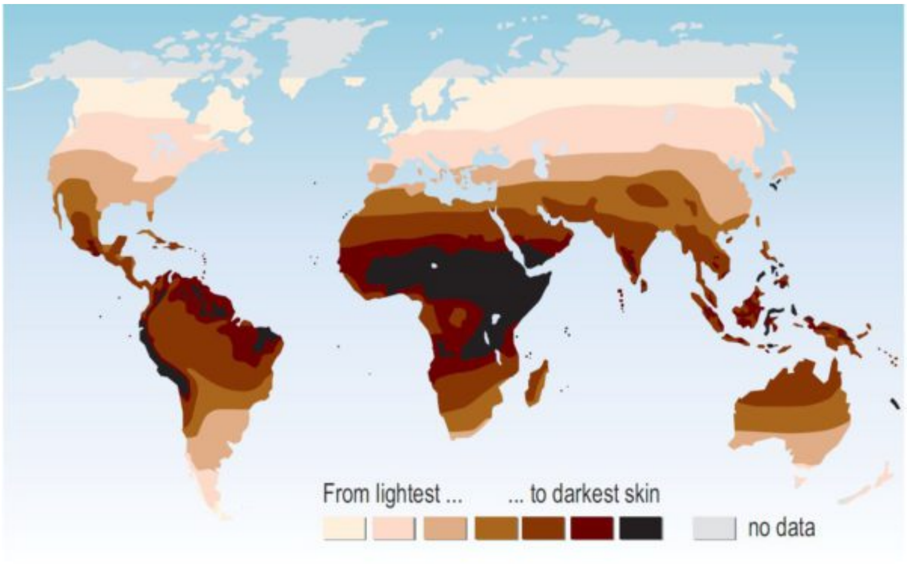

Supplementary fig. S1.1. Skin color of global indigenous peoples predicted from multiple environmental factors. Source: (Chaplin 2004); map updated in 2007.

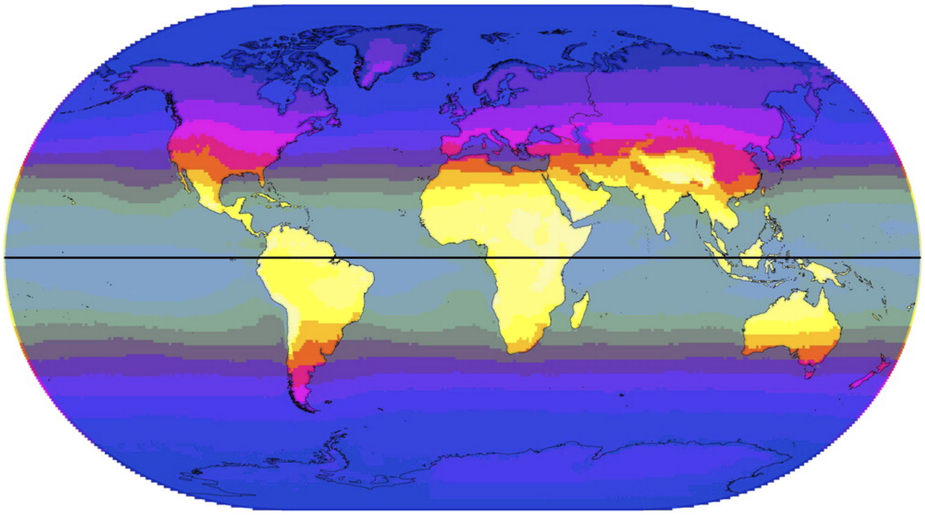

Supplementary fig. S1.2. Annual mean UVB radiation in global areas. Source: Fig. 1A in (Jablonski and Chaplin 2010). Dark color represents relatively low UVB level, while light color represents relatively high UVB level.

Supplementary table S1.1. Melanin index (MI) measurement in global populations.

Population	Sampling location	# Individuals	MI Average	MI range	Reference
Papuan	Papua New Guinea	278	74.4	-	(Norton, et al. 2006)
Negrito	Peninsular Malaysia	55	55.1	34–70	(Ang, et al. 2012)
Senoi	Peninsular Malaysia	412	45.5	28–75	(Ang, et al. 2012)
Proto-Malay	Peninsular Malaysia	50	42.2	30–61	(Ang, et al. 2012)
East Asian	Canada	334	37.8	-	(Rawofi, et al. 2017)
South Asian	India	1674	44.2	28–79	(Basu Mallick, et al. 2013)
European	Europe	469	29	20–39	(Candille, et al. 2012)
African American	USA	232	53.4	32–80	(Parra, et al. 2004)
African Caribbean	UK	173	57.8	38–80	(Parra, et al. 2004)
African	Botswana, Ethiopia, and Tanzania	1796	87.6	69.2–119.9	(Crawford, et al. 2017)
Latin American	Brazil, Colombia, Chili, Mexico, and Peru	6357	35.0	20–65	(Adhikari, et al. 2019)

Supplementary note S2. Sample collection and data preparation. World-wide population samples were obtained from public resources (Reich, et al. 2011; Henn, et al. 2012; Schlebusch, et al. 2012; Petersen, et al. 2013; Pugach, et al. 2013; Deng, et al. 2014; Ko, et al. 2014; Lazaridis, et al. 2014; Patin, et al. 2014; Aghakhanian, et al. 2015; Liu, et al. 2015; The 1000 Genomes Project Consortium 2015; Lu, et al. 2016; Mallick, et al. 2016; Mondal, et al. 2016; Pagani, et al. 2016; Lu, et al. 2017) and from our own data collection. Principal component analysis (PCA) showed that the samples clustered correspondingly to their geographical locations with little batch effect due to multiple sources of the integrated dataset (supplementary fig. S1D). We grouped the population samples collected from each tropical region (e.g., Africa, South Asia, Southeast Asia, Oceania, and the Americas) into two main groups, namely indigenous and non-indigenous populations. The indigenous populations recognized in this study were tribal populations adopting hunting, gathering, or simple agriculture. A full list of indigenous samples included in this study is provided in supplementary table S2.1. We paid special attention to some of the indigenous populations that were anthropologically identified as Negritos that displaying distinct morphological traits, such as short stature, dark pigmentation, wavy hair, and broad nose. These populations were denoted as Negrito-like (NL) indigenous populations, and those that did not match any of these descriptions were therefore grouped as non-Negrito-like (nNL) indigenous populations. Notably, as the population genetic background in Africa and the Americas are very complex, which is not in the main scope of this study, we highlighted only several indigenous populations in these two areas, and labeled all the other African and American populations as non-indigenous. In South and Southeast Asia, most of the indigenous populations have been urbanized for generations, and were thus excluded. Whole-genome sequencing samples are listed in supplementary table S2.2. Information on 50 new Orang Asli samples obtained from five populations is provided in supplementary table S2.3.

Fifty Orang Asli samples were recruited from five populations in Peninsular Malaysia. They could be assorted to three major tribes, namely Negrito (11 Bateq and 9 Mendriq), Senoi (10 Temiar and 10 CheWong) and Proto-Malay (10 Jakun). Written informed consent was obtained from all participants. Research/ethics approval and permits were obtained from the Research and Ethics Committee of Universiti Teknologi MARA [Ref no: 600-RMI (5/1/6)], the Department of Orang Asli Development (Jabatan Kemajuan Orang Asli Malaysia, JAKOA) [JHEOA.PP.30.052.Jld 5(17)], as well as the district offices, village chief, and the chairperson of

the Committee of Village Development and Security. Sample collection was also approved by the Biomedical Research Ethics Committee of Shanghai Institutes for Biological Sciences (ER-SIBS-261903).

Prior to the commencement of study, a courtesy visit was made to the headmen of each village, and the purpose of the study and procedure were explained to the headmen, in the presence of a representative from JAKOA. Approval was obtained from the district offices, village headmen, and the chairperson of the Committee of Village Development and Security. Prior to sampling, the purpose of study, sampling procedure, potential benefits and disadvantages to the community were explained to the volunteers. The volunteers were made clear with their right to withdraw the study if feel uncomfortable. Informed written consent was obtained from the volunteers aged 18 years and above. The family history, pedigree, and self-reported ethnicity were recorded via an interview using local dialect. The whole process of engagement, explanation, interview and sampling procedure were carried out in the presence of a JAKOA officer as witness. Health screening programme was conducted to the villagers during sampling and follow ups were made based on their outcomes of the health screening. Approximately 8 ml of peripheral blood was collected from the volunteers by a trained phlebotomist. All procedures were in accordance with the ethical standards of the Responsible Committee on Human Experimentation (approved by the Biomedical Research Ethics Committee of Shanghai Institutes for Biological Sciences) and the Helsinki Declaration of 1975, as revised in 2000.

Supplementary table S2.1 Groups of indigenous samples. AFR, Africa; SAS, South Asia; MSEA, Mainland Southeast Asia; ISEA, Island Southeast Asia; OCN, Oceania; AMR, America. The NL indigenous populations in Asia are indicated with "‡".

Region	Indigenous Populations	Sample Size (Indigenous/Total)	Reference
AFR	Bongo, Gui, GuiGhanaKgal, Hadza, Haiom, Himba, Hoan, Juhoan, Juhoansi, Karretjie, Khomani, Khwe, Sandawe, Taa, Tshwa, Xun, Baka, Batwa, Biaka, Mbuti, Congo Pygmies	524/1295	(Schlebusch, et al. 2012; Petersen, et al. 2013; Lazaridis, et al. 2014; Patin, et al. 2014; Mallick, et al. 2016; Pagani, et al. 2016)
SAS	Kharia, Birhor, Irula, Kusunda, Jarawa‡, Onge‡	66/808	(Lazaridis, et al. 2014; Mallick, et al. 2016; Mondal, et al. 2016)
MSEA	Jakun, Temuan, Seletar, Temiar, CheWong, MahMeri, Bateq‡, Jehai‡, Kensiu‡, Kintak‡, Lanoh‡, Mendriq‡	207/452	(Deng, et al. 2014; Aghakhanian, et al. 2015; Liu, et al. 2015), This study
ISEA	Bajo, Lebbo, Manobo, Mamanwa‡, Aeta‡, Agta‡, Batak‡	23/237	(Reich, et al. 2011; Pugach, et al. 2013; Ko, et al. 2014; Lazaridis, et al. 2014; Pagani, et al. 2016)
OCN	Aboriginal Australian, Bougainville, Papuan, Koinanbe, Kosipe	85/135	(Reich, et al. 2011; Pugach, et al. 2013; Ko, et al. 2014; Lazaridis, et al. 2014; Mallick, et al. 2016; Pagani, et al. 2016)
AMR	Cabecar, Inga, Kaqchikel, Mayan, Mixe, Mixtec, Piapoco, Pima, Ticuna, Wayuu, Chane, Guarani, Karitiana, Quechua, Surui	104/548	(Lazaridis, et al. 2014)

Supplementary table S2.2 Sample information of the next-generation sequencing (NGS) dataset. The NL and nNL indigenous populations in Asia are indicated with "‡" and "†", respectively.

Region	Population		Sample Size		Reference
AFR	ESN		99		(The 1000 Genomes Project Consortium 2015)
	GWD		113		
	LWK		99		
	MSL		85		
	YRI		108		
	Khoe-San	Juhoan_North	6	4	(Mallick, et al. 2016)
		Khomani		2	
	Rainforest hunter-gatherer	Biaka	9	2	(Mallick, et al. 2016; Pagani, et al. 2016)
		Congo_pygmies		3	
		Mbuti		4	
WES	CEU		99		(The 1000 Genomes Project Consortium 2015)
	FIN		99		
	GBR		91		
	IBS		107		
	TSI		107		
SAS	BEB		86		
	GIH		103		
	ITU		102		
	PJL		96		
	STU		102		
	Rajput		5		(Mondal, et al. 2016)

<table>
<tr><th>Region</th><th colspan="2">Population</th><th colspan="2">Sample Size</th><th>Reference</th></tr>
<tr><td rowspan="7"></td><td colspan="2">Brahmin</td><td colspan="2">10</td><td rowspan="3"></td></tr>
<tr><td colspan="2">Vellalar</td><td colspan="2">9</td></tr>
<tr><td colspan="2">Birhor†</td><td colspan="2">9</td></tr>
<tr><td colspan="2">Irula†</td><td colspan="2">12</td><td>(Mallick, et al. 2016; Mondal, et al. 2016)</td></tr>
<tr><td colspan="2">Kusunda†</td><td colspan="2">2</td><td>(Mallick, et al. 2016)</td></tr>
<tr><td rowspan="2">Andaman Negrito‡</td><td>Jarawa</td><td rowspan="2">10</td><td>4</td><td rowspan="2">(Mondal, et al. 2016)</td></tr>
<tr><td>Onge</td><td>6</td></tr>
<tr><td rowspan="7">MSEA</td><td colspan="2">Burnese</td><td colspan="2">8</td><td>(Pagani, et al. 2016)</td></tr>
<tr><td colspan="2">Vietnamese</td><td colspan="2">109</td><td>(The 1000 Genomes Project Consortium 2015; Pagani, et al. 2016)</td></tr>
<tr><td colspan="2">Jakun†</td><td colspan="2">10</td><td rowspan="5">This study</td></tr>
<tr><td colspan="2">CheWong†</td><td colspan="2">10</td></tr>
<tr><td colspan="2">Temiar†</td><td colspan="2">10</td></tr>
<tr><td rowspan="2">Malaysian Negrito‡</td><td>Mendriq</td><td rowspan="2">20</td><td>9</td></tr>
<tr><td>Bateq</td><td>11</td></tr>
<tr><td rowspan="12">ISEA</td><td colspan="2">Ami</td><td colspan="2">2</td><td rowspan="2">(Mallick, et al. 2016)</td></tr>
<tr><td colspan="2">Atayal</td><td colspan="2">1</td></tr>
<tr><td colspan="2">Luzon</td><td colspan="2">2</td><td rowspan="7">(Pagani, et al. 2016)</td></tr>
<tr><td colspan="2">Murut</td><td colspan="2">8</td></tr>
<tr><td colspan="2">Dusun</td><td colspan="2">8</td></tr>
<tr><td colspan="2">Igorot</td><td colspan="2">8</td></tr>
<tr><td colspan="2">Visayan</td><td colspan="2">2</td></tr>
<tr><td colspan="2">Bajo†</td><td colspan="2">4</td></tr>
<tr><td colspan="2">Lebbo†</td><td colspan="2">4</td></tr>
<tr><td rowspan="3">Philippine Negrito‡</td><td>Aeta</td><td rowspan="3">9</td><td>3</td><td rowspan="5">(Pagani, et al. 2016)</td></tr>
<tr><td>Agta</td><td>3</td></tr>
<tr><td>Batak</td><td>3</td></tr>
<tr><td rowspan="3">OCN</td><td colspan="2">Koinanbe†</td><td colspan="2">3</td></tr>
<tr><td colspan="2">Kosipe†</td><td colspan="2">3</td></tr>
<tr><td colspan="2">Papuan†</td><td colspan="2">15</td><td>(Mallick, et al. 2016)</td></tr>
<tr><td rowspan="4">EAS</td><td colspan="2">Tibetan</td><td colspan="2">38</td><td>(Lu, et al. 2016)</td></tr>
<tr><td colspan="2">Dai</td><td colspan="2">97</td><td>(The 1000 Genomes Project Consortium 2015; Mallick, et al. 2016)</td></tr>
<tr><td colspan="2">Han</td><td colspan="2">298</td><td>(The 1000 Genomes Project Consortium 2015; Lu, et al. 2016; Lu, et al. 2017)</td></tr>
<tr><td colspan="2">JPT</td><td colspan="2">104</td><td rowspan="5">(The 1000 Genomes Project Consortium 2015)</td></tr>
<tr><td rowspan="4">AMR</td><td colspan="2">CLM</td><td colspan="2">94</td></tr>
<tr><td colspan="2">MXL</td><td colspan="2">64</td></tr>
<tr><td colspan="2">PEL</td><td colspan="2">85</td></tr>
<tr><td colspan="2">PUR</td><td colspan="2">104</td></tr>
</table>

Supplementary table S2.3 Information of the 50 Malaysian Orang Asli samples.

Sample ID	Gender	Depth		Sample ID	Gender	Depth	
		Raw	Filtered			Raw	Filtered
CheWong (from Kuala Gandah, Lanchang, Pahang)							
AAGC031001	female	35.46	32.48	AAGC031002	male	33.40	31.12
AAGC031003	female	31.66	29.20	AAGC031004	female	37.01	33.75
AAGC031005	female	35.25	32.19	AAGC031006	female	31.42	28.72
AAGC031007	female	35.27	32.26	AAGC031008	female	36.12	33.00
AAGC031009	female	33.29	30.33	AAGC031010	female	32.78	30.20
Temiar (from Kuala Betis, Gua Musang, Kelantan)							
AAGC031011	female	37.70	34.70	AAGC031012	female	34.90	32.16
AAGC031013	male	38.82	36.01	AAGC031014	female	32.18	29.52
AAGC031015	male	37.23	34.50	AAGC031016	female	41.73	37.73
AAGC031017	male	41.76	38.09	AAGC031018	male	40.10	37.27
AAGC031019	female	36.28	33.41	AAGC031020	male	41.47	38.28
Jakun (from Tasik Chini, Chini, Pahang)							
AAGC031021	female	39.43	35.88	AAGC031022	female	29.19	25.92
AAGC031023	male	33.35	30.14	AAGC031024	female	28.83	25.42
AAGC031025	female	29.24	25.91	AAGC031026	female	29.65	26.25
AAGC031027	male	34.34	30.66	AAGC031028	male	32.38	29.01
AAGC031029	female	38.79	34.11	AAGC031030	male	31.16	28.11
Mendriq (from Kuala Lah, Gua Musang, Kelantan)							
AAGC031031	male	32.83	28.98	AAGC031032	female	37.29	32.83
AAGC031033	female	31.01	27.55	AAGC031034	female	33.35	29.26
AAGC031035	female	29.40	25.85	AAGC031036	male	32.04	28.93
AAGC031037	female	31.36	27.52	AAGC031038	male	29.64	26.70
AAGC031039	male	29.53	26.63				
Bateq (from Sungai Aring, Gua Musang, Kelantan)							
AAGC031040	male	33.66	30.12	AAGC031041	male	29.55	26.44
AAGC031042	female	36.71	33.56	AAGC031043	male	41.71	38.89
AAGC031044	male	42.94	39.83	AAGC031045	male	40.64	37.88
AAGC031046	male	29.34	22.37	AAGC031047	female	29.21	21.78
AAGC031048	female	32.97	30.27	AAGC031049	female	37.32	34.23
AAGC031050	female	36.00	32.95				

DNA was extracted from the peripheral blood using QIAGEN DNeasy Blood & Tissue Kit. DNA concentration was checked using the NanoDrop 2000 (Thermo Fisher Scientific), and sheared with Covaris S220 Sonicator (Covaris) to target of 500–600 base pairs (bp) average size. Fragmented DNA was purified using Sample Purification Beads (Illumina). Adapter-ligated

libraries were prepared with the TruSeq Nano DNA Sample Prep Kits (Illumina) according to Illumina-provided protocol. DNA concentrations of the resulting sequencing libraries were measured with the Qubit 2.0 fluorometer dsDNA HS Assay (Thermo Fisher Scientific). Quantities and sizes of the resulting sequencing libraries were analyzed using Agilent BioAnalyzer 2100 (Agilent). The libraries were used in cluster formation on an Illumina cBOT cluster generation system with HiSeq X HD PE Cluster Kits (Illumina). The 50 samples were sequenced at 30× coverage (ranging in 28.8 – 42.9×) for 150 bp paired-end reads, using Illumina Hiseq X10 version 2.5 in Wuxi NextCODE at Shanghai. Each sample was run on a unique lane with at least 90 GB PF data. The reads data were filtered to ensure that 80% of the bases achieved at least a base quality score of 30.

The raw reads were aligned to the reference GRCh37 human genome using the 'mem' algorithm in Burrows-Wheeler Aligner (BWA) version 0.7.10-r789 (Li and Durbin 2010), and then the output files were converted to the binary format (BAM) using SAMtools version 0.1.19-44428 (Li, et al. 2009) and sorted using Picard toolkit version 1.117 (http://broadinstitute.github.io/picard/). Reads with mapping quality (MAPQ) < 20 and those potential duplicated reads produced during library construction were eliminated by SAMtools version 0.1.19-44428 (Li, et al. 2009) and Picard toolkit version 1.117, respectively. We realigned the indels using IndelRealigner from GATK version 3.2-0-g289df4b (McKenna, et al. 2010), with the 1000 Genomes Project Phase I indels as the reference dataset. Finally, the base quality score recalibration (BQSR) was performed to adjust the base quality score bias from the sequencer, using the 1000 Genomes Phase I indels and dbSNP (version 147) as the training datasets.

Variant calling was performed using HaplotypeCaller of GATK version 3.2-0-g289df4b (McKenna, et al. 2010; Depristo, et al. 2011). Variant positions are reported in hg19/GRCh37 coordinates. For the SNPs located around the indels, we applied *de novo* local assembly to improve the variant calling accuracy. Then, the variants quality score recalibration (VQSR) module in GATK was used to filter the raw SNPs and indels separately. To avoid potential duplications or deletions, only variants with depth in 1/3−2.5× of the mean read depth were retained. The mapping quality (MQ) ≥ 30 was also used as a criterion to select variants covered by high mapping quality reads.

We then applied a procedure of universal mask developed by Li et al. to the genome-wide variants (Mallick, et al. 2016), which is a sample-dependent mask that removes complex regions (~600 Mb in length) where variant calling can be challenging. We further filtered out the multi-allelic SNPs, and those with missing rate over 20% in any one of the five Orang Asli populations. Finally, 51,722,790 SNPs were reserved for further analyses. We further inferred the sample relatedness based on genome-wide variants using KING version 2.1.2 (Manichaikul, et al. 2010), and identified some first-degree relatedness (supplementary fig. S2.1).

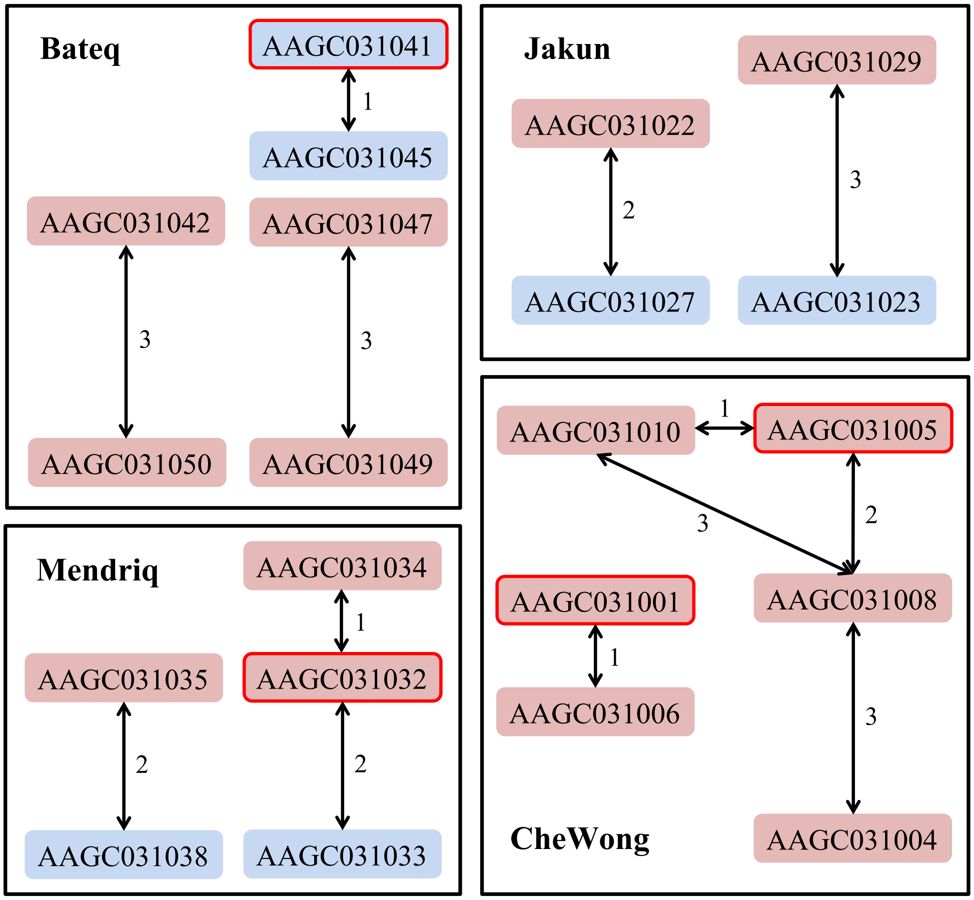

Supplementary fig. S2.1. Relatedness among the sequenced samples in each Orang Asli population. Graphical representation of the kinship relationships (first, second and third degrees) among Orang Asli males (light blue) and females (pink). Samples highlighted with red border are removed in the subsequent analyses to ensure that all the samples in each Orang Asli population are unrelated.

Haplotype phasing was carried out independently for 12 different sources of datasets using Shapeit2 (Delaneau, et al. 2011). For the SNP-array data, we first merged the data generated by the sample platform (Affymetrix or Illumina) by keeping the shared SNPs. Then we filtered out sites with genotype missing rate > 10% in the SNP-array data and those with genotype missing

rate > 5% in the sequencing data. Parameters were set as default in phasing. Detailed phasing strategy for each dataset can be found in supplementary table S2.4.

Supplementary table S2.4 Phasing strategies for different datasets. Dataset 1–10 are phased independently. Dataset 11 and 12 are phased data downloaded from Pagani et al. (Pagani, et al. 2016) and the 1000 Genomes Project database (The 1000 Genomes Project Consortium 2015), respectively. Dataset 1–7 are phased using a reference dataset (indicated by "*"), including 103,517,266 SNPs of 3,792 samples from the SGDP data, the 1000 Genomes data, 50 Orang Asli sequences, 90 Han Chinese sequences (Lu, et al. 2016; Lu, et al. 2017), and 38 Tibetan sequences (Lu, et al. 2016).

Dataset	Platform	Populations	Sample Size	#sites	
				Before phasing	After phasing
1*	Illumina	amaXhosa, Juhoan, Xun, XunV	48	918,462	916,958
2*	Illumina	BakaCam, BakaGab Bakiga, Batwa, BongoGabE, BongoGabS, NzebiGab, NzimeCam	260	905,002	885,731
3*	Illumina	Biaka, Mbuti	34	643,902	643,571
4*	Illumina	GuiGhanaKgal, Juhoansi, Karretjie, Khomani, Khwe, Nama, SEBantu, SWBantu, XunD	180	2,266,602	2,134,134
5*	Illumina	MoroccoN, MoroccoS, SaharaOCC	52	702,904	702,431
6*	Illumina	Bateq, CheWong, Jakun, Jehai, MahMeri, Mendriq, Seletar	134	2,287,796	2,156,590
7*	Affymetrix	Bateq, Jehai, Kensiu, Kintak, Lanoh, Mendriq, Malay, Temiar, Temuan	84	857,426	856,694
8	Affymetrix	Ami, Atayal, Bunun, Paiwan, Pingpu, Puyuma, Rukai, Saisiat, Tsou, Papuan, Polynesia, Dravidian, Alor, Besemah, Borneo, Fiji, Flores, Hiri, Mamanwa, Manobo, Roti, Semende, Ternate, Timor	229	762,309	762,309
9	Affymetrix	The Human Origin populations	2227	593,124	593,124
10	NGS	Han Chinese, Tibetan, Birhor, Irula, Rajput, UBR, Vellalar, Onge, Jarawa, and the SGDP populations	269	46,712,206	46,557,907
11	NGS	The EGDP populations	181	-	42,971,058
12	NGS	The 1000 Genomes populations	2054	-	77,818,345

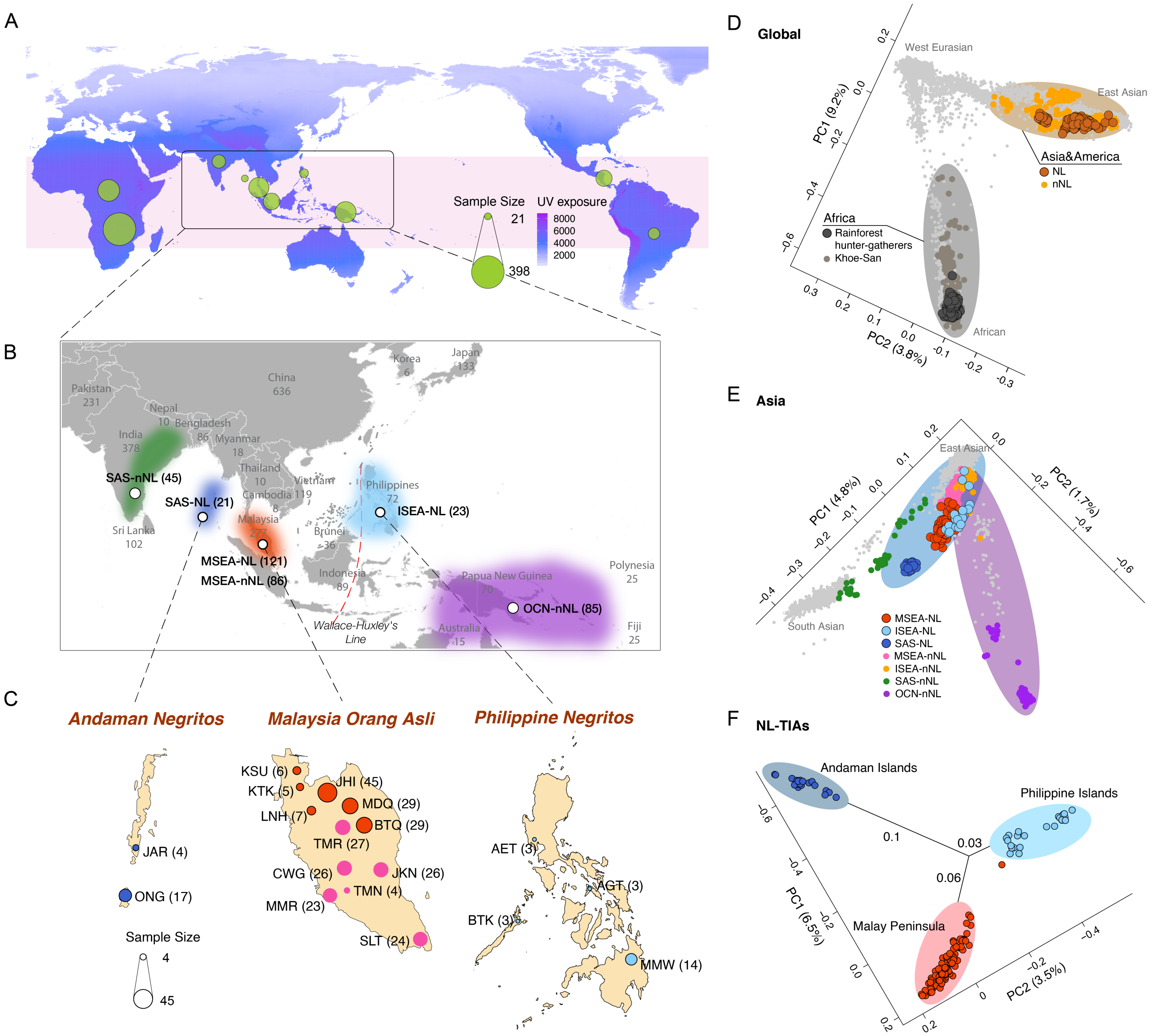

Supplementary fig. S1. Sample information and principal component analysis (PCA). (*A*) Location of the tropical indigenous samples analyzed in this study. The ultraviolet (UV) data were downloaded from https://www.ufz.de/gluv/. (*B*) Asian samples (including Oceanic samples) used in this study, with tropical indigenous Asian (TIA) sample locations shaded. Red dashed line denotes the Wallace-Huxley's biogeographical line. (*C*) Negrito-like (NL) TIAs from South and Southeast Asia, and non-Negrito-like (nNL) Orang Asli in Peninsular Malaysia. The maps in (*A*), (*B*), and (*C*) were drawn using *R* according to the coordinates downloaded from https://www.esrichina.com.cn/. (*D*), (*E*), and (*F*) present the PCA plots of (*D*) 6,059 world-wide samples, (*E*) 2,346 Asian samples, and (*F*) 165 NL-TIA samples, in correspondence with (*A*), (*B*), and (*C*), respectively. In (*F*), there is an outlier Mendriq Negrito sample from Malaysia overlapping with the Philippine Negritos due to recent admixture of Malay and Mendriq Negritos. The first two PCs are shown in each plot.

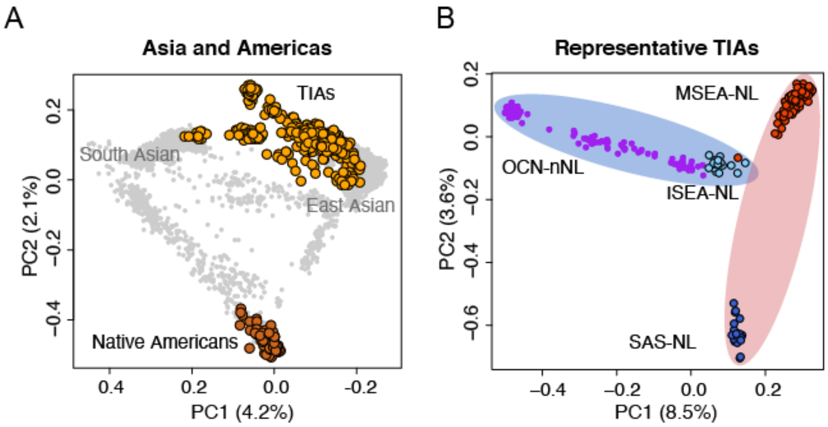

Supplementary fig. S2. Principal component analysis (PCA) of (*A*) Asia and American samples and (*B*) NL-TIAs and Papuans. These plots are complements of supplementary fig. S1D–F. They are performed using 9,452 SNPs in 3,150 individuals and in 287 individuals, respectively, based on the same set of SNPs as that used in supplementary fig. S1D–F.

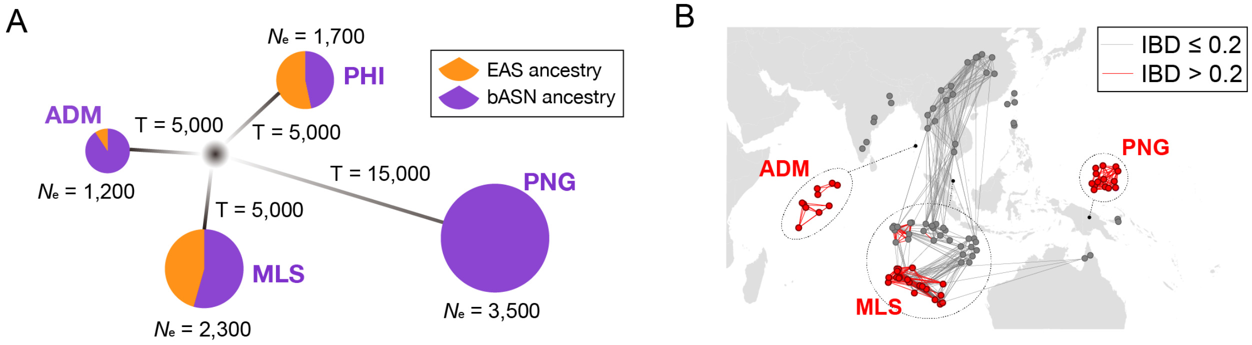

Supplementary fig. S3. Demographic estimation of the Malaysian Negritos, the Andaman Negritos, the Philippine Negritos, and the Papuans. (*A*) Estimation of effective population size (N_e), divergence time, and ancestry admixture. Each pie represents a population, with the size proportional to N_e, and the branch length denoting drift time (in years) for each population since divergence. N_e was estimated for each population by applying the LD-based method (McEvoy, et al. 2011). A total of 10 replicates were conducted to minimize the potential bias, and 10 individuals were random sampled from each population. Further, we estimated the divergence time in generations by $2N_eF_{ST}$, in which the global F_{ST} was measured using genome-wide variants. (*B*) Estimation of the identical-by-descent (IBD) tracks. IBD sharing was estimated between unrelated individuals within each population or across populations, using PLINK version 1.9 (Purcell, et al. 2007). Each dot represents an individual. The solid lines connecting two individuals suggest identified IBD sharing.

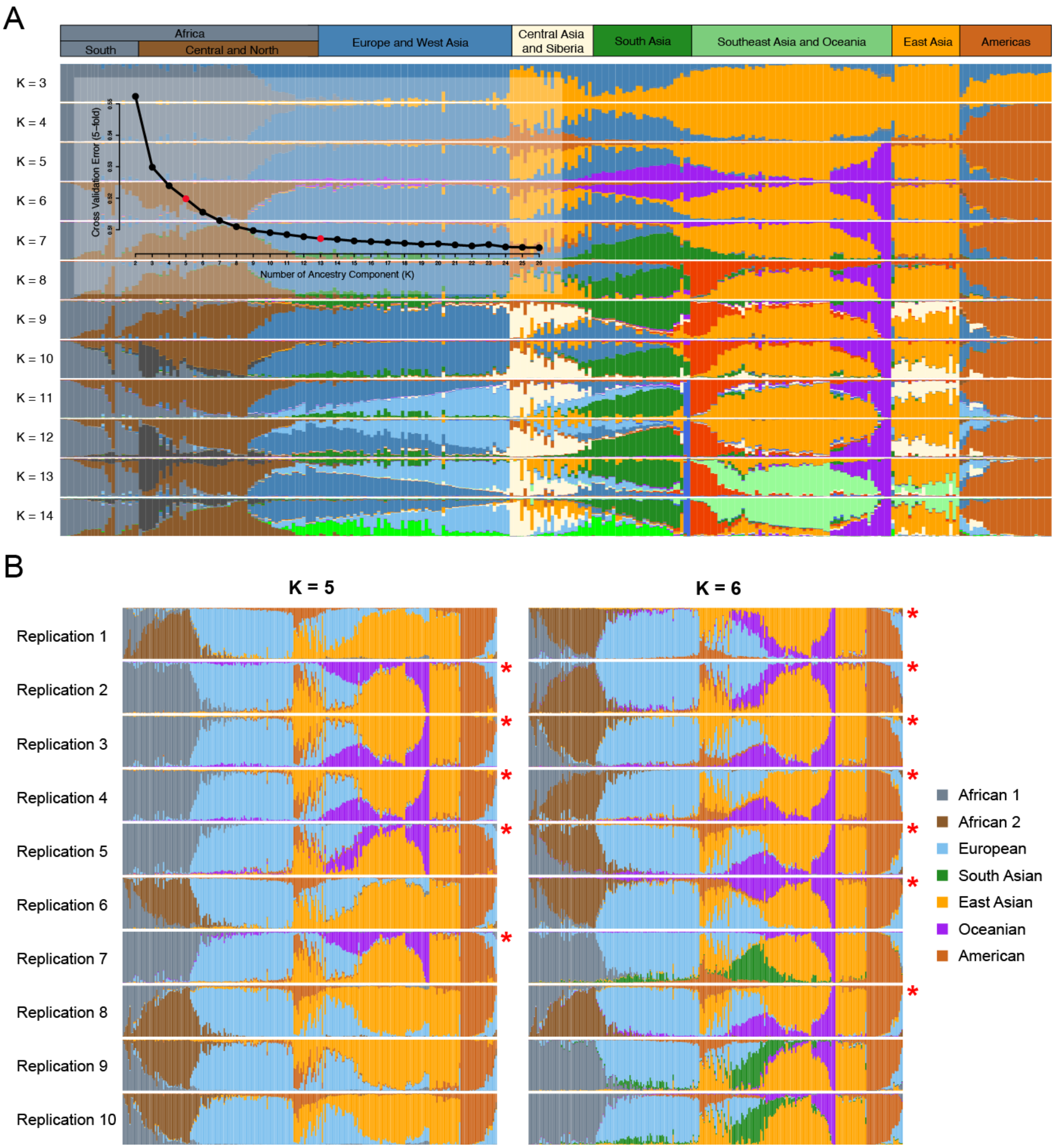

Supplementary fig. S4. Unsupervised ADMIXTURE analysis (*A*) from *K* = 3 to *K* = 14 and (*B*) replicated analyses with random seeds for *K* = 5 to *K* = 6, performed on all the 6,059 world-wide samples with default parameters. The bASN ancestry component (noted as Oceanian) is supported by eight (indicated by asterisks) of the ten replications.

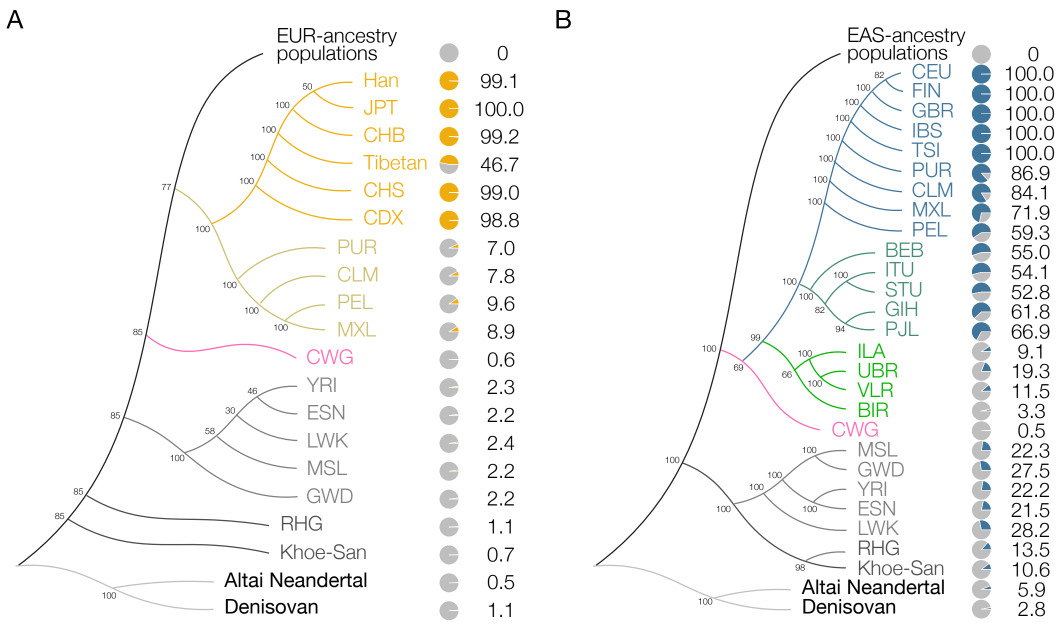

Supplementary fig. S5. Maximum-likelihood trees for (*A*) the European-specific variants and (*B*) the East Asian-specific variants, in comparison with that for the Papuan-specific variants in fig. 2C. Tree are constructed using Phylip version 3.2 and visualized using the *ggtree* package implemented in *R*. Sharing of the European-specific or East Asian-specific alleles (%) in each population was shown on the right of each branch.

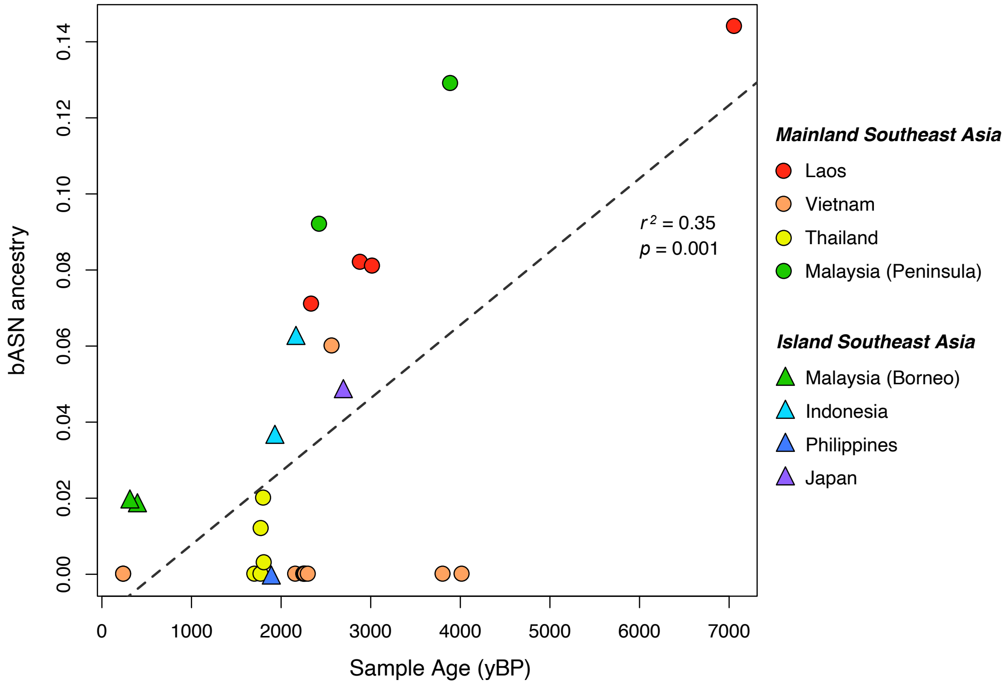

Supplementary fig. S6. Positive correlation between the bASN ancestry inferred by ADMIXTURE projection and age of the Neolithic Southeast Asian samples.

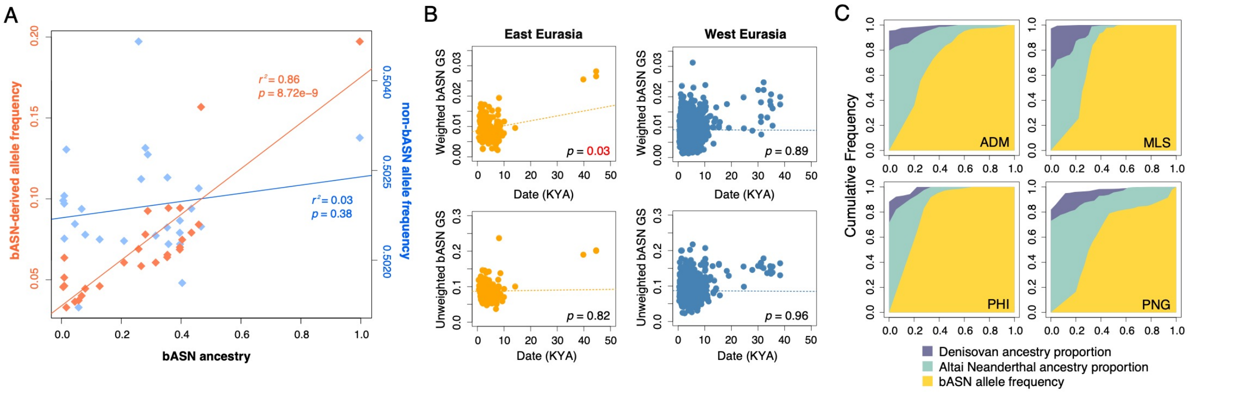

Supplementary fig. S7. The bASN allele analysis. (*A*) Correlation between the bASN-derived alleles (red) or randomly selected non-bASN-derived allele frequency (blue) and the bASN ancestry across populations. Each triangle represents the mean allele frequency against the bASN ancestry proportion in a population. (*B*) Positive correlation between the genetic score (GS) of the bASN-derived alleles and the age of ancient human samples in Eurasia. (*C*) Cumulative pattern of the bASN-derived allele frequency and the archaic ancestry proportion at the bASN loci in each of the four bASN-enriched population.

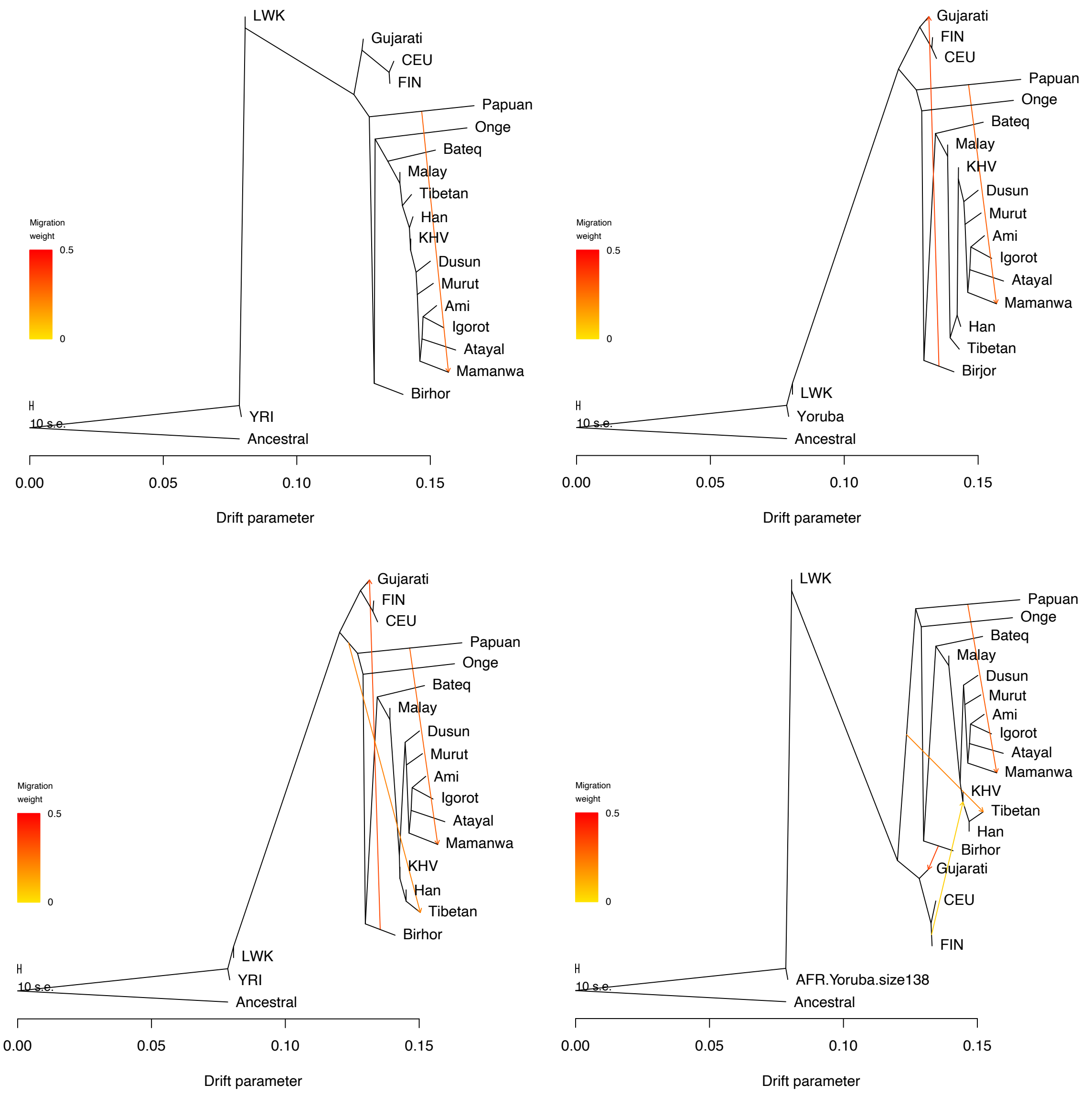

Supplementary fig. S8. TreeMix analysis with 1–4 migrations. Inferred ancestral genome information from the 1000 Genomes Project was used as the outgroup. The scale bar shows ten units of standard error (s.e.), and the amount of drift is plotted along the x-axis. Gene flow from Papuan to Philippine Negrito (represented by Mamanwa) is consistently shown in the four plots.

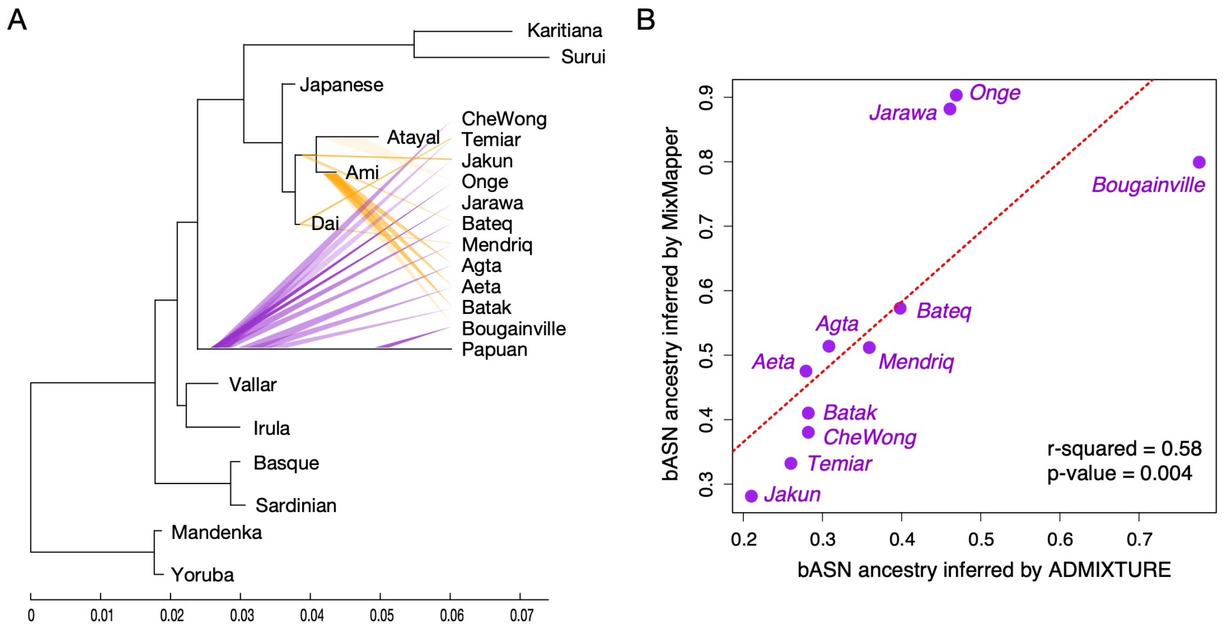

Supplementary fig. S9. (*A*) Ancestry source inference using MixMapper. The admixed populations were inferred as two-way admixture. The shaded ranges indicated 95% confidence intervals of the branching positions, and the transparency represented the admixture proportions. (*B*) Positive correlation between the bASN ancestry inferred by ADMIXTURE and that inferred by MixMapper.

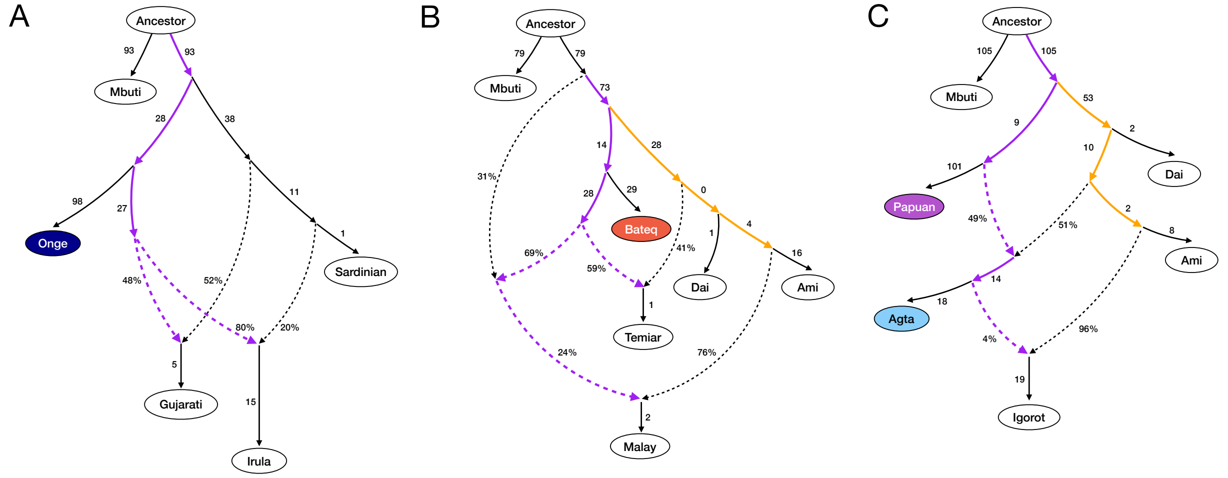

Supplementary fig. S10. Admixture graphs inferred for (*A*) South Asian, (*B*) Mainland Southeast Asian, and (*C*) Island Southeast Asian. The purple arrows indicate the path for the bASN ancestry, and the yellow arrows indicate the modern East Asian ancestry. Lengths and admixture proportions are shown for the branches.

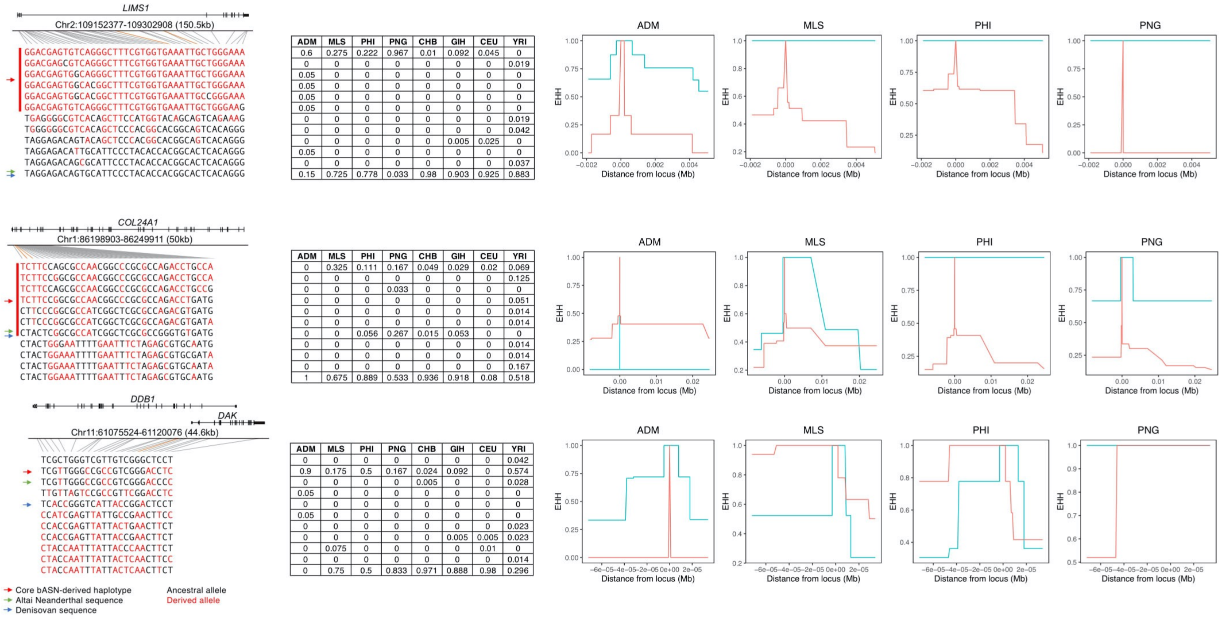

Supplementary Fig. S11. Examples of adaptive bASN loci. Haplotype sharing and extended haplotype homozygosity (EHH) at the bASN loci in *LIMS1*, *COL24A1*, and *DDB1*/*DAK*. EHH plots are shown at rs7604136, rs313723, and rs12290116 as focal SNPs in these three genes, respectively.

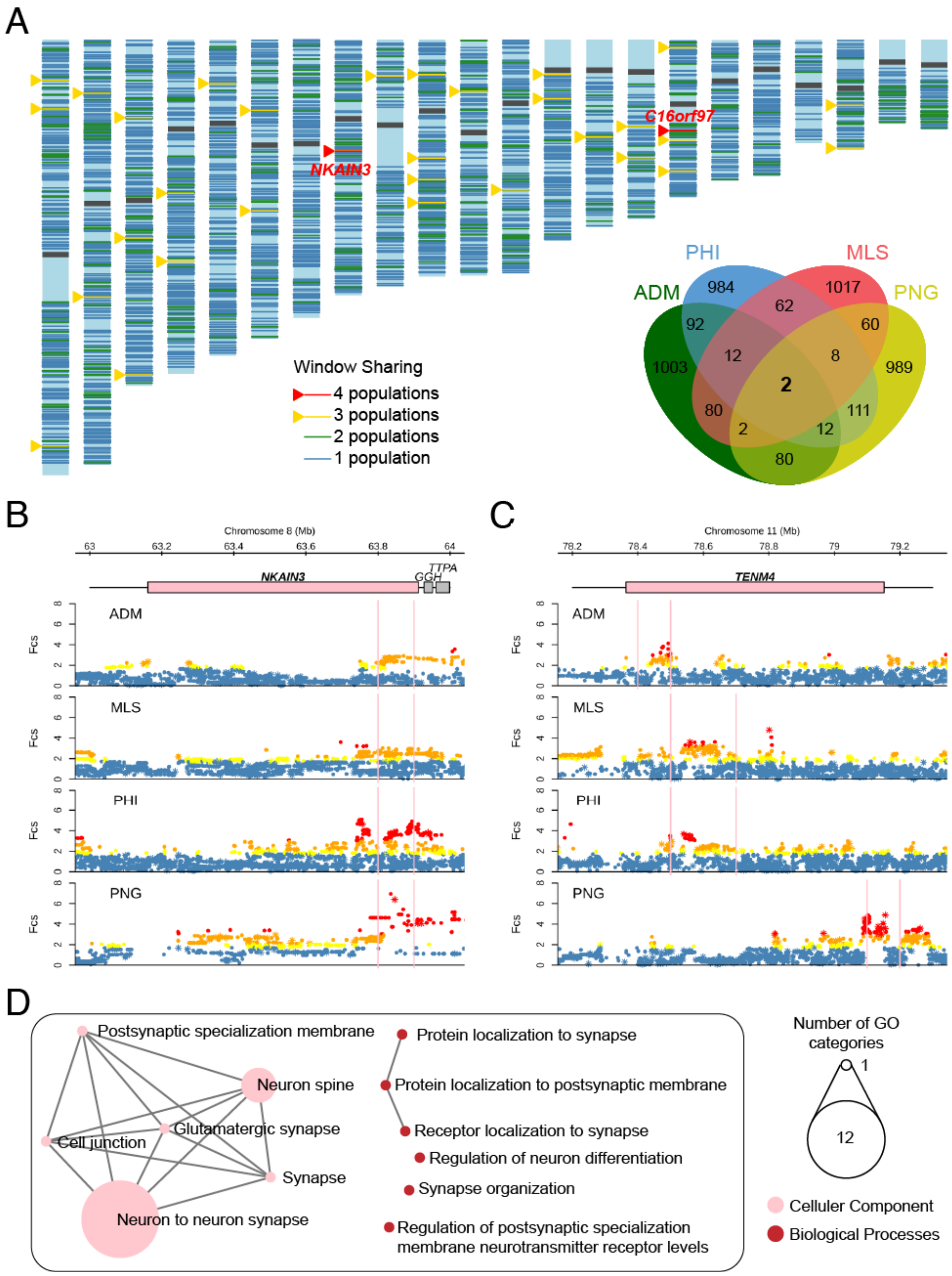

Supplementary fig. S12. Signals of selective sweeps in the representative TIA populations. (*A*) Sharing of the sweep signals among the four representative TIA populations. Colored triangles on the chromosomes indicate genomic regions that are common to at least three populations. (*B*) Selective sweep signals at *NKAIN3*, which are identical across the four populations. (*C*) Selective sweep signals at *TENM4*, which are different across the four populations. In (*B*) and (*C*), the signal windows are indicated by the pink vertical lines. Significance level of F_{CS} for each SNP is indicated by colored dots: red, $p < 0.01$; orange, $0.01 < p < 0.05$; yellow, $0.05 < p < 0.1$. (*D*) Gene Ontology (GO) enrichment of the adaptive genes shared by at least two populations. The GO categories were summarized and visualized using REVIGO (Supek, et al. 2011).

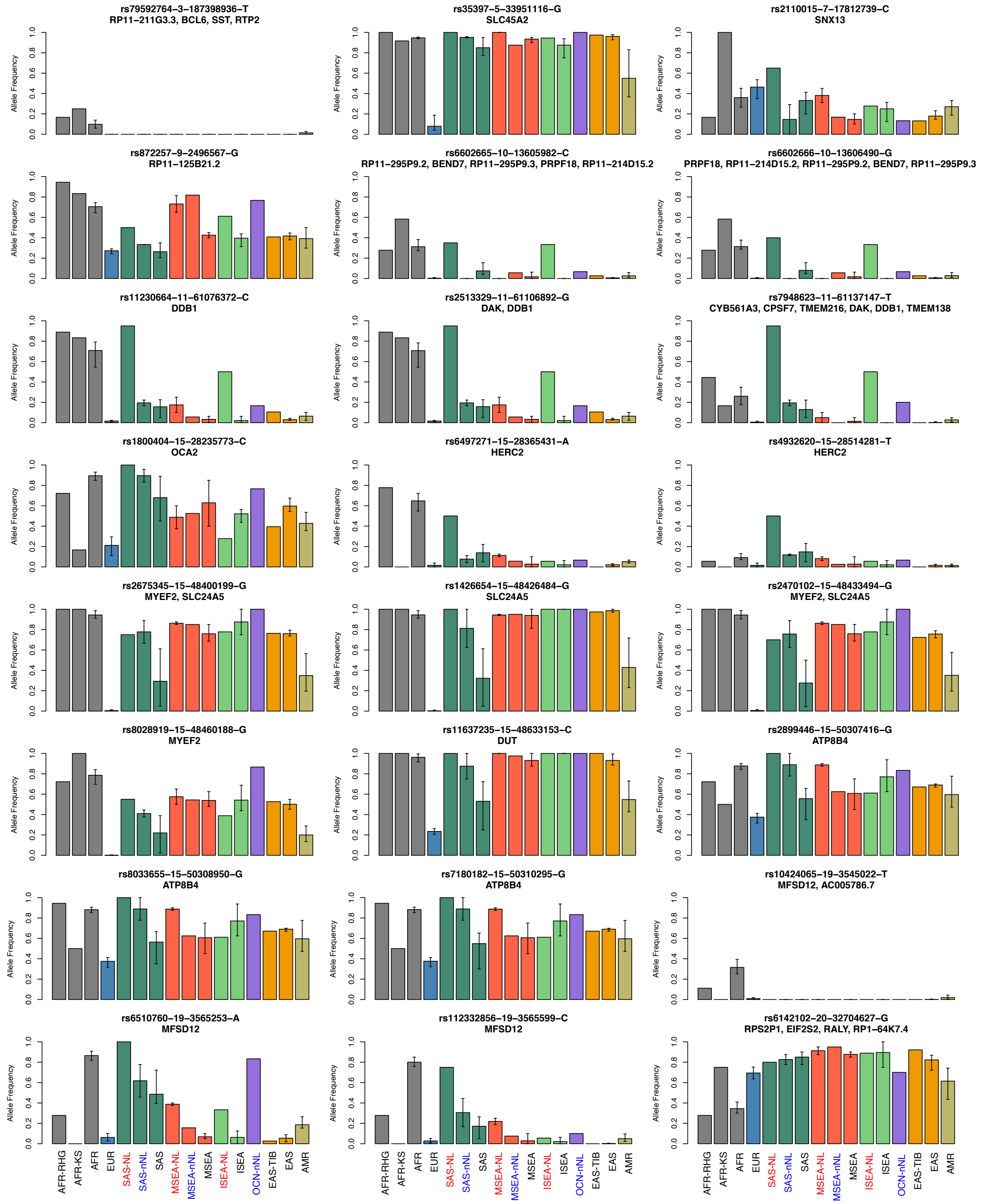

Supplementary fig. S13. Allele frequency of the African-reported dark-pigmentation alleles in global populations. Populations with samples size < 8 were not included in this analysis. The bars plot shows the mean and range of pigmentary alleles (if the population size is > 1 in the group) in global populations. The NL-TIA and nNL-TIA groups are labeled with red and blue fonts, respectively. RHG, rainforest hunter-gatherer; KS, Khoe-San; NL, Negrito-like; nNL, non-Negrito-like; TIB, Tibetan.

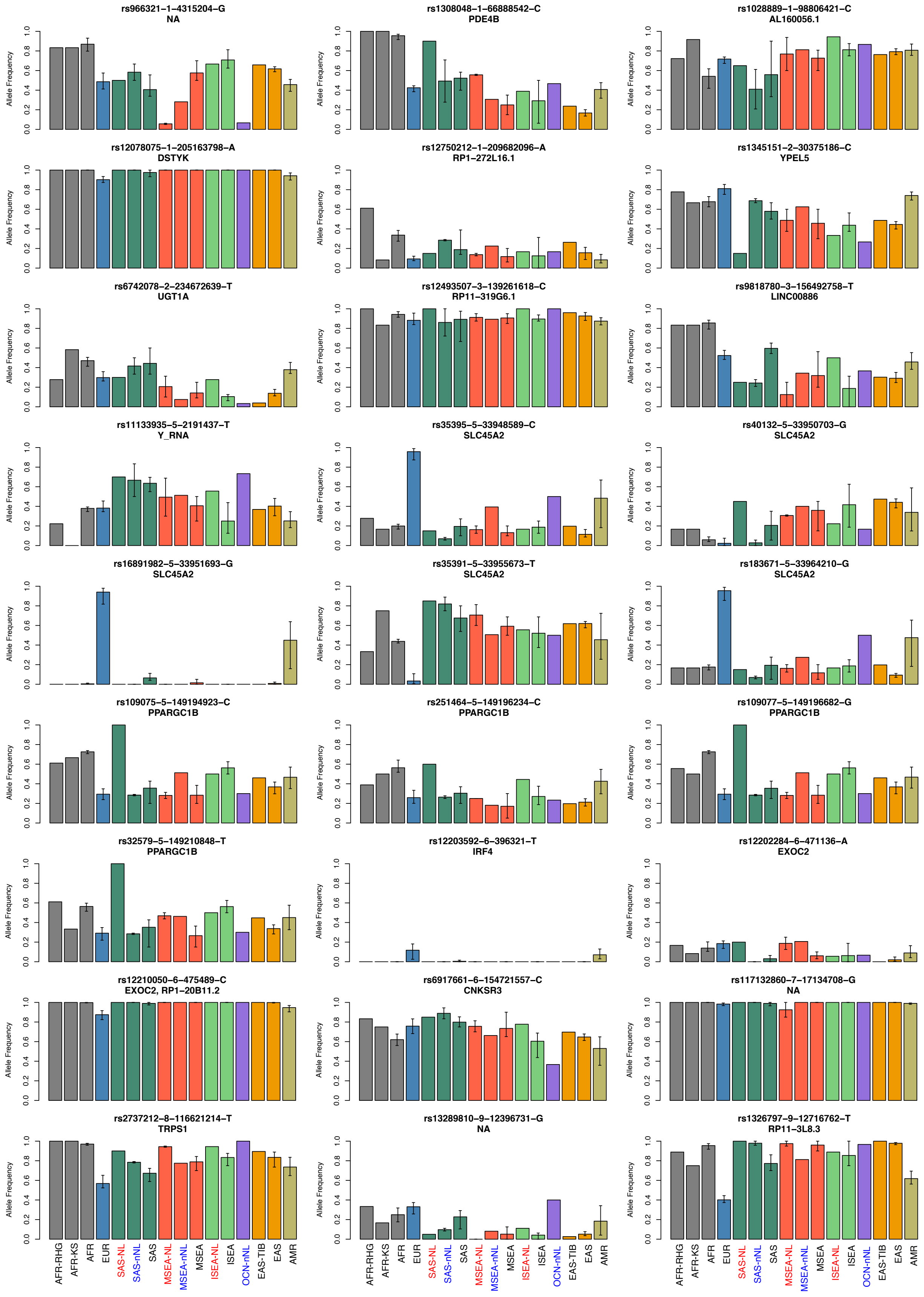
rs966321–1–4315204–G
NA
rs1308048–1–66888542–C
PDE4B
rs1028889–1–98806421–C
AL160056.1
rs12078075–1–205163798–A
DSTYK
rs12750212–1–209682096–A
RP1–272L16.1
rs1345151–2–30375186–C
YPEL5
rs6742078–2–234672639–T
UGT1A
rs12493507–3–139261618–C
RP11–319G6.1
rs9818780–3–156492758–T
LINC00886
rs11133935–5–2191437–T
Y_RNA
rs35395–5–33948589–C
SLC45A2
rs40132–5–33950703–G
SLC45A2
rs16891982–5–33951693–G
SLC45A2
rs35391–5–33955673–T
SLC45A2
rs183671–5–33964210–G
SLC45A2
rs109075–5–149194923–C
PPARGC1B
rs251464–5–149196234–C
PPARGC1B
rs109077–5–149196682–G
PPARGC1B
rs32579–5–149210848–T
PPARGC1B
rs12203592–6–396321–T
IRF4
rs12202284–6–471136–A
EXOC2
rs12210050–6–475489–C
EXOC2, RP1–20B11.2
rs6917661–6–154721557–C
CNKSR3
rs117132860–7–17134708–G
NA
rs2737212–8–116621214–T
TRPS1
rs13289810–9–12396731–G
NA
rs1326797–9–12716762–T
RP11–3L8.3
Allele Frequency
0.0
0.2
0.4
0.6
0.8
1.0
AFR-RHG
AFR-KS
AFR
EUR
SAS-NL
SAS-nNL
SAS
MSEA-NL
MSEA-nNL
MSEA
ISEA-NL
ISEA
OCN-nNL
EAS-TIB
EAS
AMR

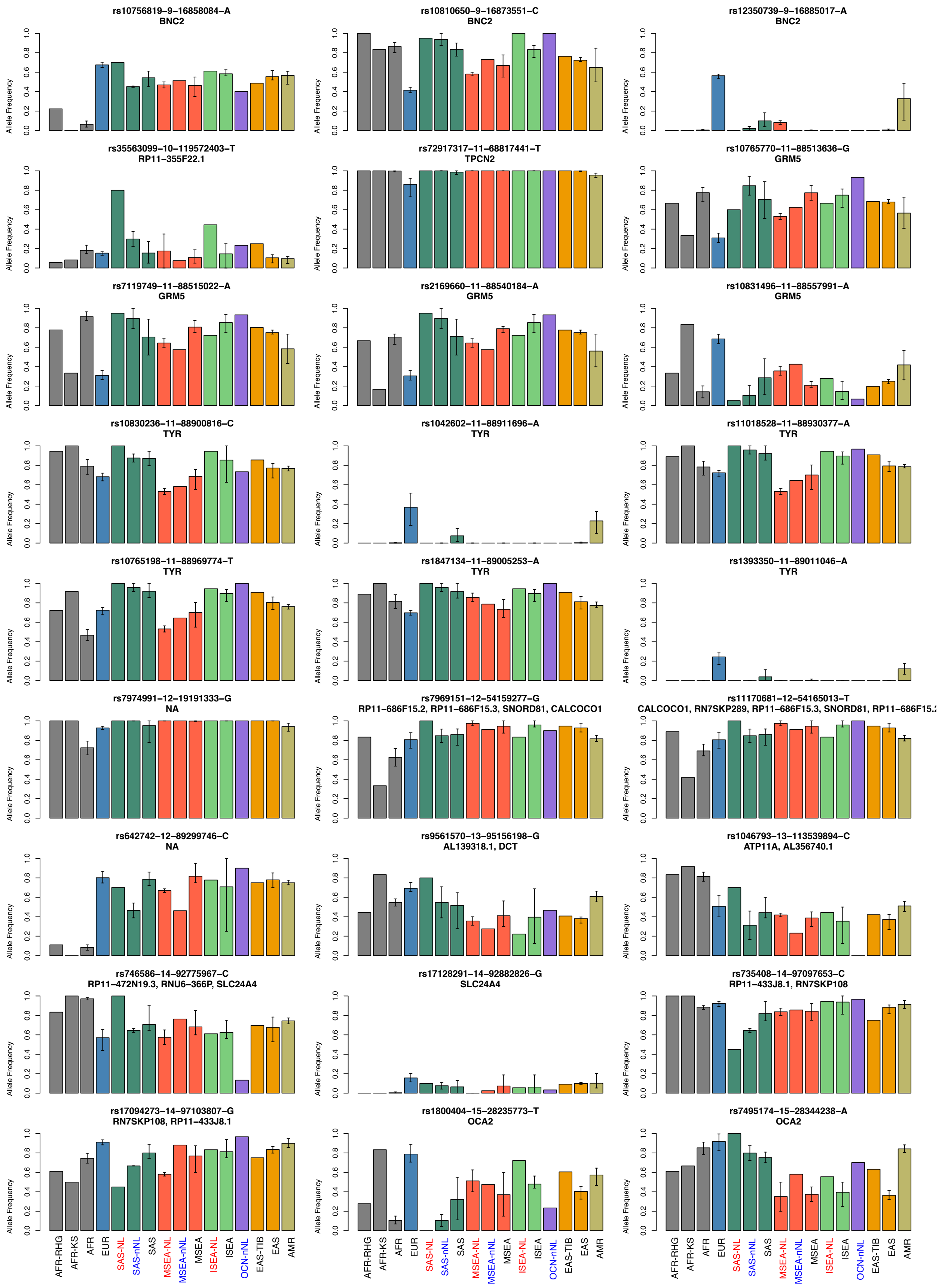
rs10756819–9–16858084–A
BNC2
rs10810650–9–16873551–C
BNC2
rs12350739–9–16885017–A
BNC2
rs35563099–10–119572403–T
RP11–355F22.1
rs72917317–11–68817441–T
TPCN2
rs10765770–11–88513636–G
GRM5
rs7119749–11–88515022–A
GRM5
rs2169660–11–88540184–A
GRM5
rs10831496–11–88557991–A
GRM5
rs10830236–11–88900816–C
TYR
rs1042602–11–88911696–A
TYR
rs11018528–11–88930377–A
TYR
rs10765198–11–88969774–T
TYR
rs1847134–11–89005253–A
TYR
rs1393350–11–89011046–A
TYR
rs7974991–12–19191333–G
NA
rs7969151–12–54159277–G
RP11–686F15.2, RP11–686F15.3, SNORD81, CALCOCO1
rs11170681–12–54165013–T
CALCOCO1, RN7SKP289, RP11–686F15.3, SNORD81, RP11–686F15.
rs642742–12–89299746–C
NA
rs9561570–13–95156198–G
AL139318.1, DCT
rs1046793–13–113539894–C
ATP11A, AL356740.1
rs746586–14–92775967–C
RP11–472N19.3, RNU6–366P, SLC24A4
rs17128291–14–92882826–G
SLC24A4
rs735408–14–97097653–C
RP11–433J8.1, RN7SKP108
rs17094273–14–97103807–G
RN7SKP108, RP11–433J8.1
rs1800404–15–28235773–T
OCA2
rs7495174–15–28344238–A
OCA2
Allele Frequency
0.0 0.2 0.4 0.6 0.8 1.0
AFR-RHG AFR-KS AFR EUR SAS-NL SAS-nNL SAS MSEA-NL MSEA-nNL MSEA ISEA-NL ISEA OCN-nNL EAS-TIB EAS AMR

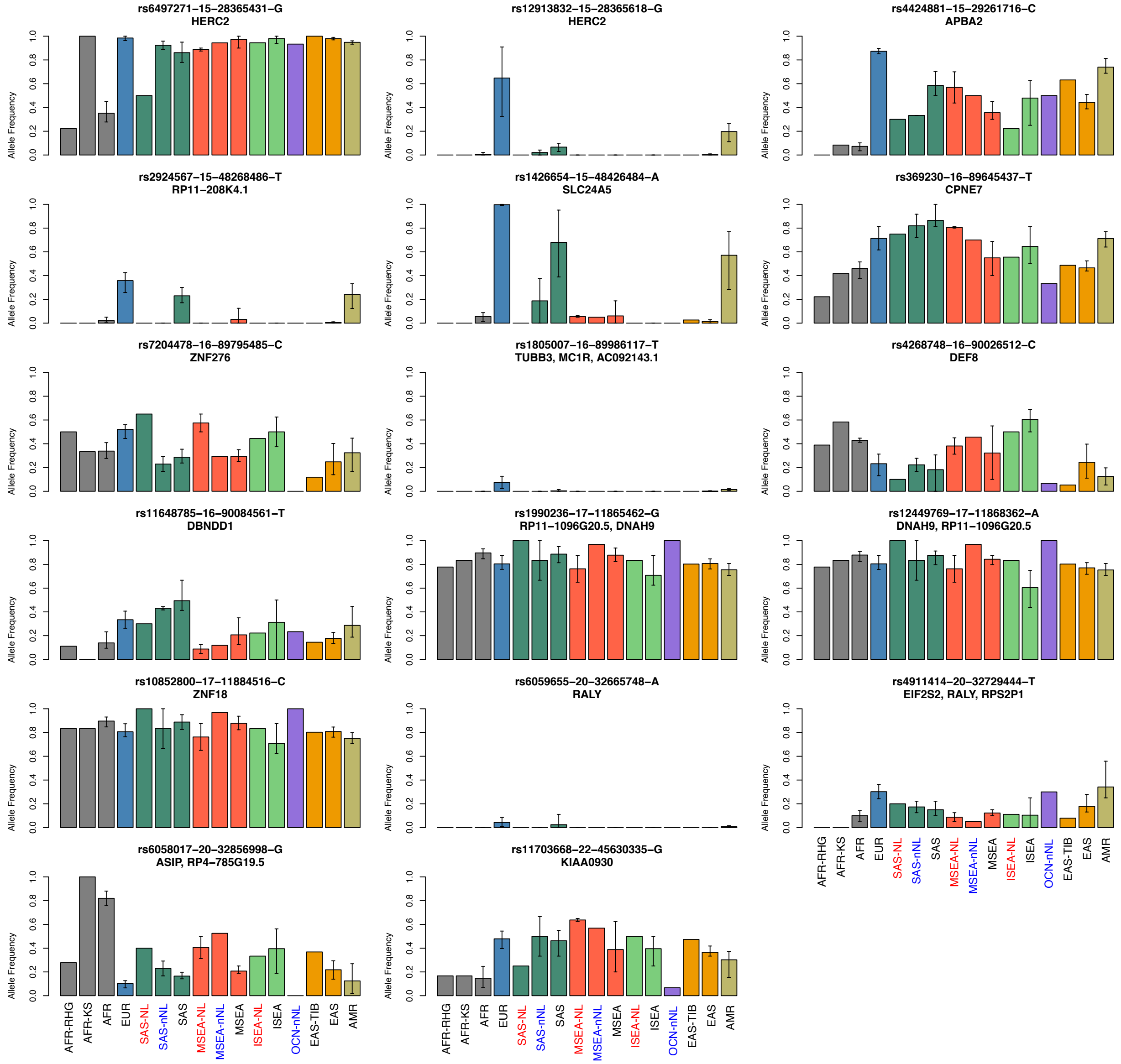

Supplementary fig. S14. Allele frequency of each European-reported light pigmentation allele in global populations. Populations with samples size < 8 were not included in this analysis. The bars plot shows the mean and range of pigmentary alleles (if the population size is > 1 in the group) in global populations. The NL-TIA and nNL-TIA groups are labeled with red and blue fonts, respectively. RHG, rainforest hunter-gatherer; KS, Khoe-San; NL, Negrito-like; nNL, non-Negrito-like; TIB, Tibetan.

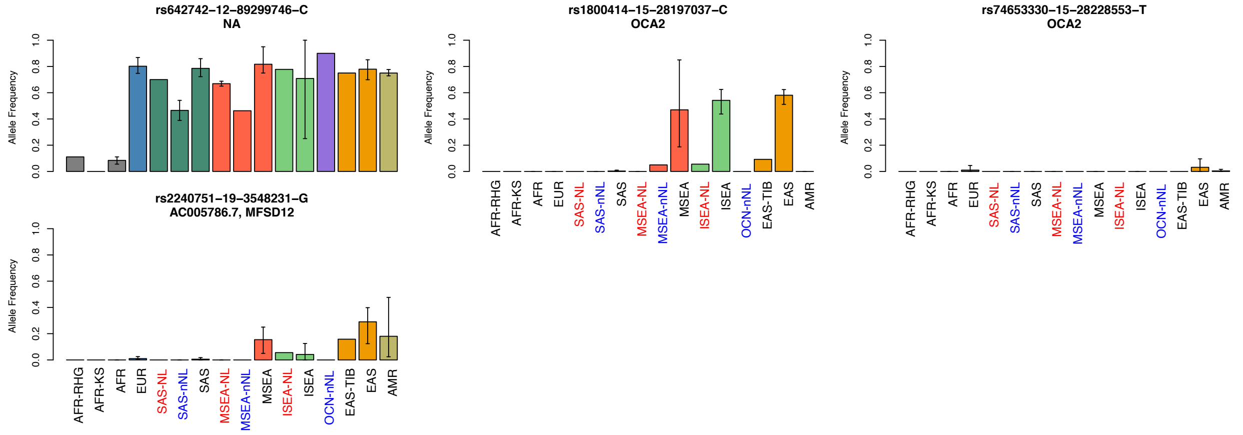

Supplementary fig. S15. Allele frequency of the East Asian-reported light-pigmentation allele in global populations. Populations with samples size < 8 were not included in this analysis. The bars plot shows the mean and range of pigmentary alleles (if the population size is > 1 in the group) in global populations. The NL-TIA and nNL-TIA groups are labeled with red and blue fonts, respectively. RHG, rainforest hunter-gatherer; KS, Khoe-San; NL, Negrito-like; nNL, non-Negrito-like; TIB, Tibetan.

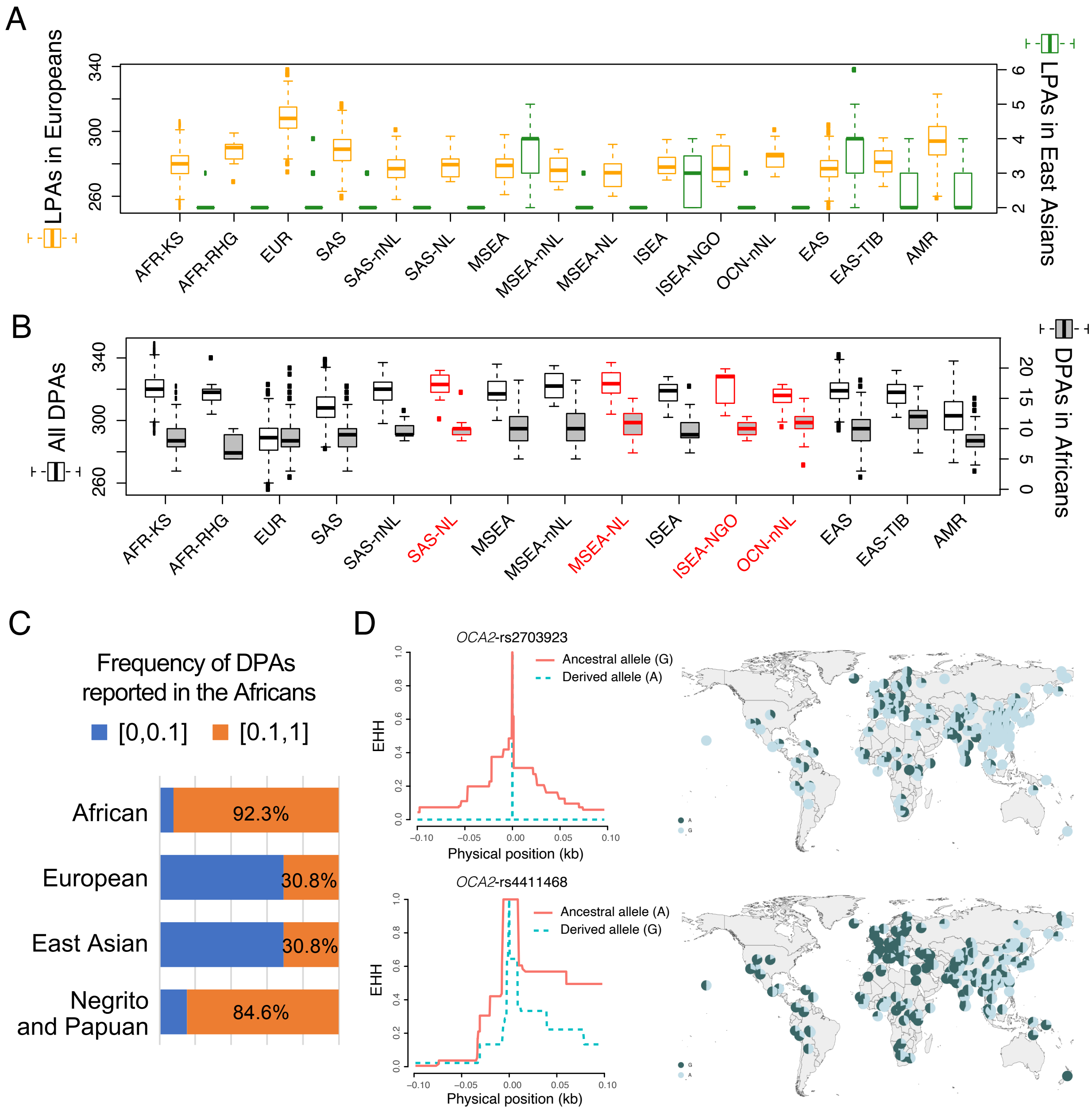

Supplementary fig. S16. Accumulation of the pigmentary alleles in global populations. (*A*) Count of light pigmentation alleles (LPAs) reported in Eurasians in each population. (*B*) Count of dark pigmentation alleles (DPAs) in each population. Representative TIA populations are highlighted. (*C*) Frequency of the DPAs reported in Africans. (*D*) Selective sweep signal at *OCA2*. Extended haplotype homozygosity (EHH) and global distribution of allele frequency at rs2703923 in the Philippine Negritos and at rs28003335 in the Papuans. The allele frequency maps are downloaded from http://pggsnv.org.

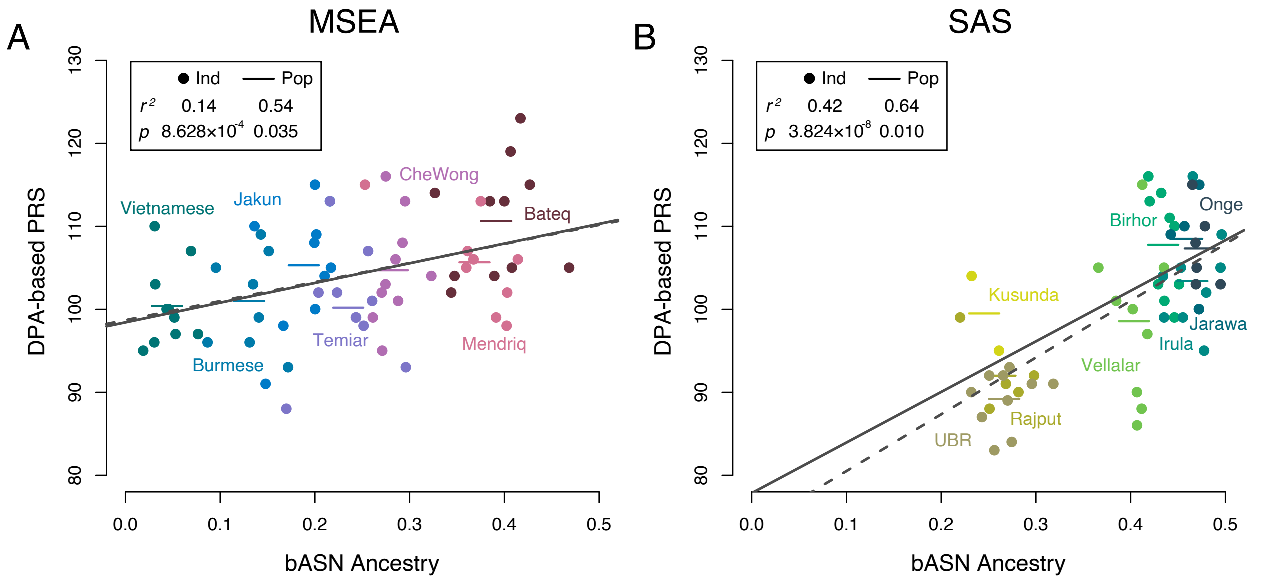

Supplementary fig. S17. Polygenic risk score (PRS) calculated based on all the dark pigmentation alleles (DPAs) reported in Africans, Europeans, and East Asians are positively correlated with the bASN ancestry. Each dot represents an individual, and the short horizontal line indicates the mean level across samples in a population. The grey dashed lines are linear regressions on individuals, and the grey solid lines are those on populations. MSEA, mainland Southeast Asia; SAS, South Asia.

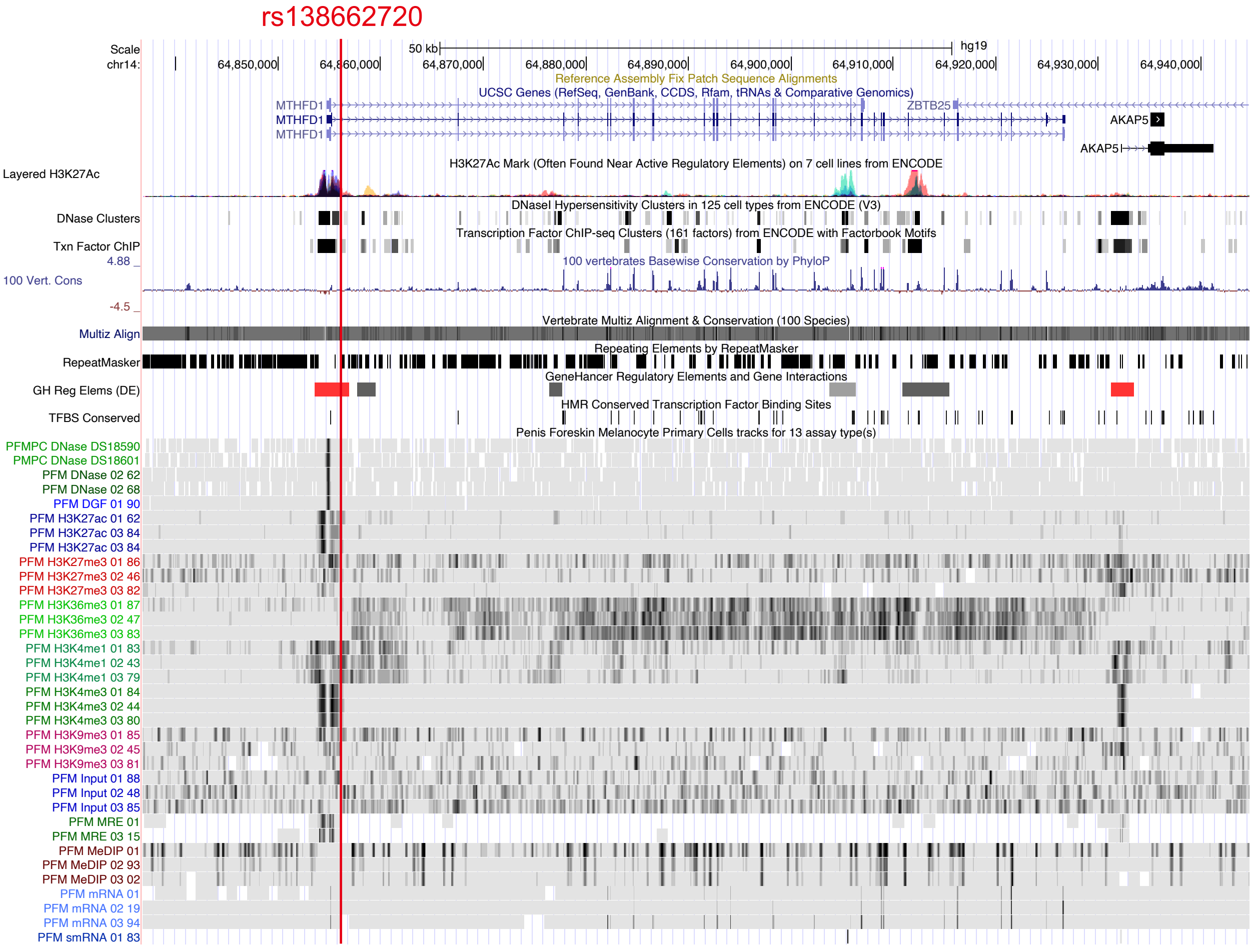

Supplementary fig. S18. Annotation of *MTHFD1*-rs138662720 using UCSC genome browser.

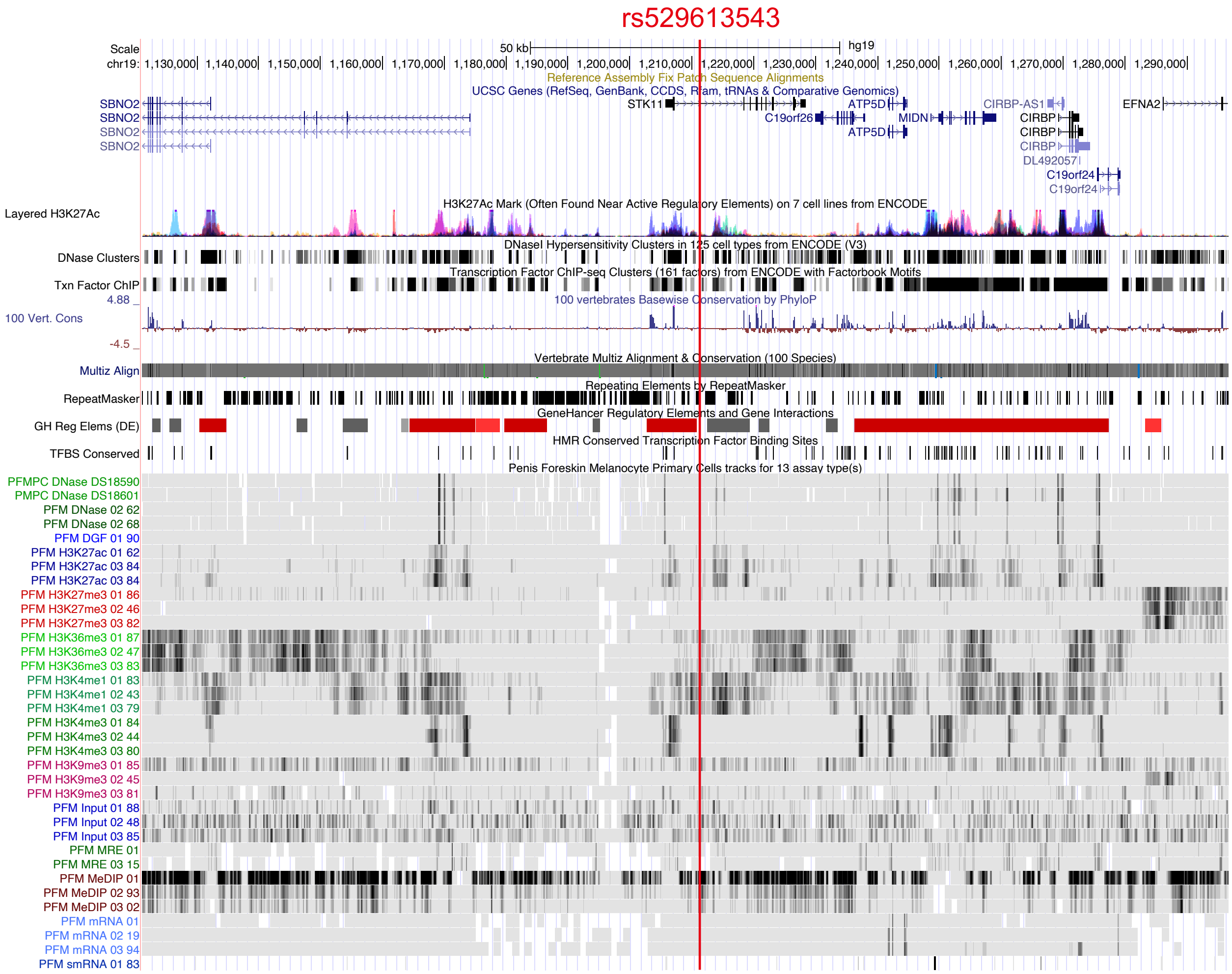

Supplementary fig. S19. Annotation of *STK11*-rs529613543 using UCSC genome browser.

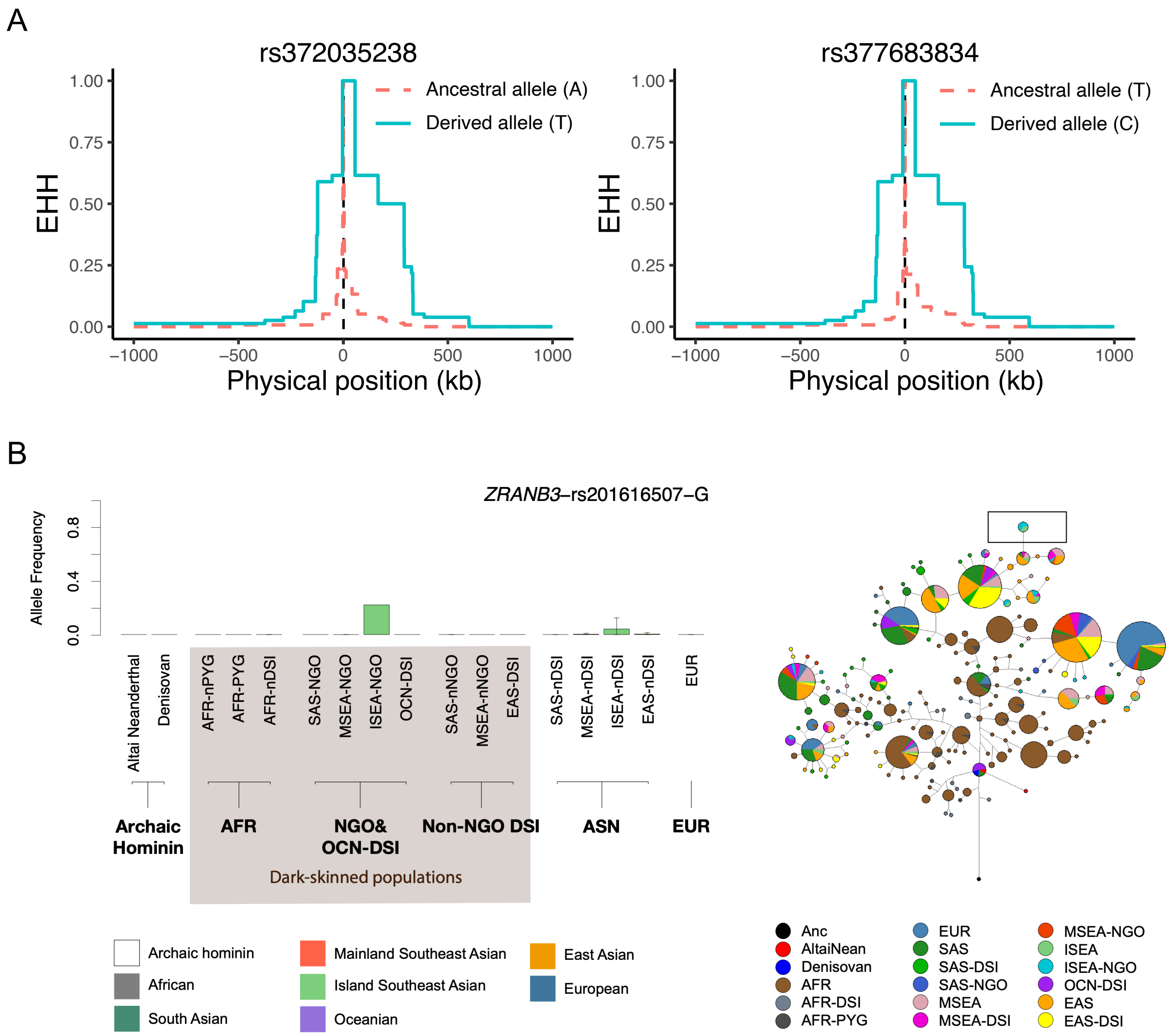

Supplementary fig. S20. Hard sweeps of population-specific *de novo* mutations. (*A*) Extended haplotype homozygosity (EHH) in *USH2A* in the Papuans. These plots show the decay of haplotype homozygosity with increasing distance from the core SNPs on the Papuan haplotypes in *USH2A*. The x-axis is the chromosome position of SNPs spanning 2 Mb around the core SNPs, and the y axis is the probability that two chromosomes are homozygous at all SNPs for the entire interval from the core region to certain distance. EHH = 0 means all extended haplotypes are different, while EHH = 1 indicates that all extended haplotypes are the same. In our plots, the green lines represent the decay of homozygosity of chromosomes carrying the derived allele at the core SNPs, while the red lines signify the decay of homozygosity on chromosomes bearing the ancestral core allele. (*B*) Adaptive signal at *ZRANB3*-rs201616507. Haplotypes carrying the target alleles are framed in black-edged boxes.

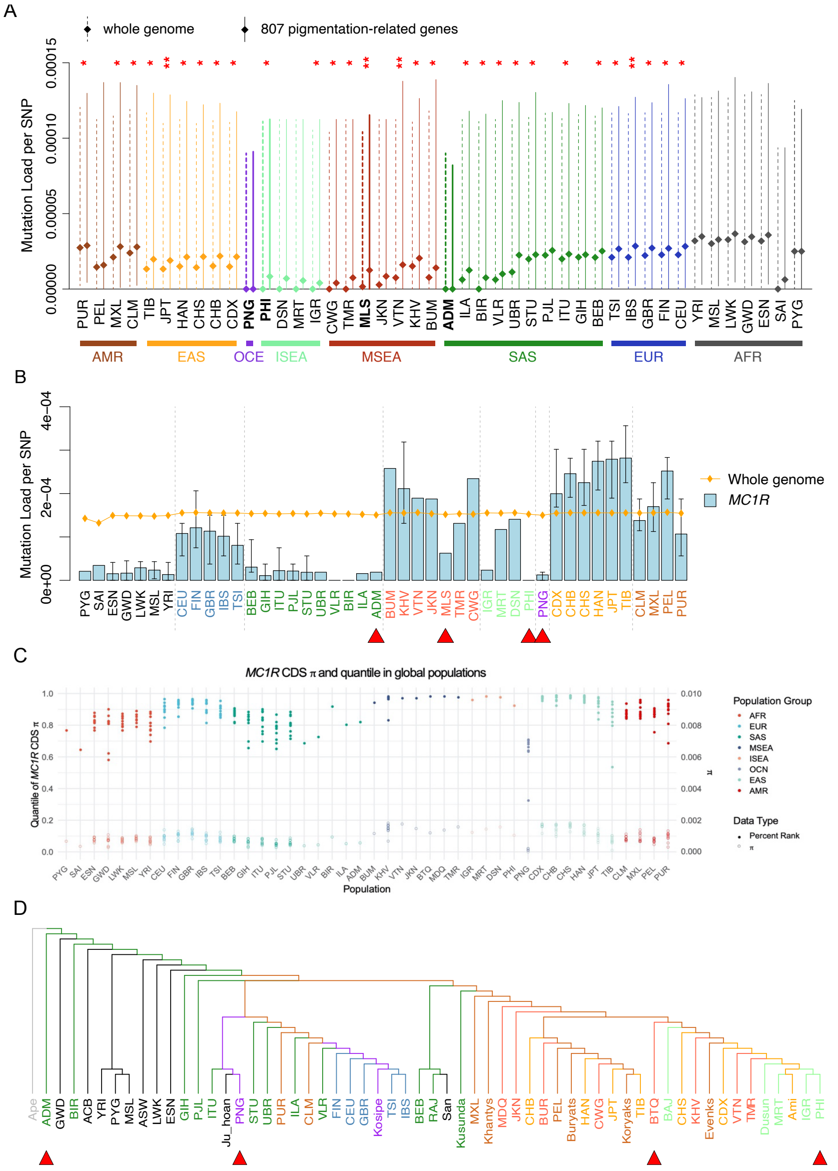

Supplementary fig. S21. Testing for purifying selection at *MC1R*. (*A*) Mutation load of pigmentary genes in global populations. Significant smaller mutation load of the pigmentary loci than that of the remaining genes are indicated with asterisks, based on the one-sided Wilcoxon's rank-sum test. "*": $0.01 < p < 0.05$; "**", $p < 0.01$. (*B*) Mutation load in *MC1R*. For populations with sample size > 10, 10 replicated analyses were performed by randomly sampling 10 individuals from each population. The error bars indicate the minimum and maximum mutation load of the replicants. Representative TIA populations are indicated with red triangles. (*C*) Nucleotide diversity (π) of *MC1R* in world-wide populations. Circles indicate π of *MC1R* coding sequence (CDS); dots represent quantiles among selected background CDS regions. There are 171 background CDS regions with similar length to that of *MC1R* major transcript ENST00000555147 (Ensembl GRCh37) (difference smaller than 20%). For populations with sample size > 10, we conducted 10 replication analyses by randomly selected 10 samples in each replicate. Colors illustrate the continental populations. (*D*) Phylogeny of *MC1R* in global populations constructed using the maximum likelihood method. The between-population distance was measured using F_{ST}. Representative TIA populations in Asia are indicated with red triangles.

Supplementary table S1. Mitochondrial DNA and Y-chromosome haplogroup frequency in global populations. Re-estimated results from the public data are indicated by "*"; others are collected from literatures. KS, Khoe-San; RHG, rainforest hunter-gatherers; NL, Negrito-like; nNL, non-Negrito-like.

Supplementary table S2. Genomic regions affected by the bASN ancestry. f_{mean}, average bASN-derived allele frequency; f_{max}, maximum bASN-derived allele frequency, obtained from the bASN-allele frequency block consisting at least five bASN-derived alleles.

Supplementary table S3. Priori pigmentary genes collected from literatures. These genes are collected from literatures, GWAS catalogue, pigmentation-related pathways (e.g., KEGG pathway and Gene Ontology). For each gene, we show the source of collection (Y, Yes; N, No), and the number of effect variants reported, separated by a slash.

Supplementary table S4. A full list of pigmentation-associated alleles reported in literatures. Ancestral alleles are indicated with "*"; Genes located ≤50 kb upstream/downstream to the intergenic variants are underlined; Protein-coding genes are labeled with bold fonts. Key variants are highlighted in blue, adapted from Table 1 in Rocha et al. (Rocha 2020). Allele frequency in global populations is calculated for the dark pigmentation allele (DPA).

Supplementary table S5. Comparing the allele sharing among the TIAs for the putative DPAs vs. non-pigmentary alleles that are missing in the non-indigenous Eurasians. AF, allele frequency; OR, odds ratio. The significant *p*-values are indicated with asterisks.

(see Supplementary_tables.xlsx for supplementary table S1–5)

References

Adhikari K, Mendoza-Revilla J, Sohail A, Fuentes-Guajardo M, Lampert J, Chacon-Duque JC, Hurtado M, Villegas V, Granja V, Acuna-Alonzo V, et al. 2019. A GWAS in Latin Americans highlights the convergent evolution of lighter skin pigmentation in Eurasia. Nat Commun 10:358.

Aghakhanian F, Yunus Y, Naidu R, Jinam T, Manica A, Hoh BP, Phipps ME. 2015. Unravelling the genetic history of Negritos and indigenous populations of Southeast Asia. Genome Biology and Evolution 7:1206-1215.

Ang KC, Ngu MS, Reid KP, Teh MS, Aida ZS, Koh DX, Berg A, Oppenheimer S, Salleh H, Clyde MM, et al. 2012. Skin color variation in Orang Asli tribes of Peninsular Malaysia. PLoS One 7:e42752.

Armstrong BK, Kricker A. 2001. The epidemiology of UV induced skin cancer. J Photochem Photobiol B 63:8-18.

Barrows DP. 1910. The Negrito and allied types in the Philippines. American Anthropologist 12:358-376.

Basu Mallick C, Iliescu FM, Mols M, Hill S, Tamang R, Chaubey G, Goto R, Ho SY, Gallego Romero I, Crivellaro F, et al. 2013. The light skin allele of SLC24A5 in South Asians and Europeans shares identity by descent. PLoS Genet 9:e1003912.

Brenner M, Hearing VJ. 2008. The protective role of melanin against UV damage in human skin. Photochem Photobiol 84:539-549.

Candille SI, Absher DM, Beleza S, Bauchet M, McEvoy B, Garrison NA, Li JZ, Myers RM, Barsh GS, Tang H, et al. 2012. Genome-wide association studies of quantitatively measured skin, hair, and eye pigmentation in four European populations. PLoS One 7:e48294.

Chaplin G. 2004. Geographic distribution of environmental factors influencing human skin coloration. Am J Phys Anthropol 125:292-302.

Chaplin G, Jablonski NG. 1998. Hemispheric difference in human skin color. Americal Journal of Physical Anthropology 107:221-224.

Crawford NG, Kelly DE, Hansen MEB, Beltrame MH, Fan S, Bowman SL, Jewett E, Ranciaro A, Thompson S, Lo Y, et al. 2017. Loci associated with skin pigmentation identified in African populations. Science 358:eaan8433.

de Gruijl FR, van Kranen HJ, Mullenders LH. 2001. UV-induced DNA damage, repair, mutations and oncogenic pathways in skin cancer. J Photochem Photobiol B 63:19-27.

Delaneau O, Marchini J, Zagury JF. 2011. A linear complexity phasing method for thousands of genomes. Nat Methods 9:179-181.

Deng L, Hoh BP, Lu D, Fu R, Phipps ME, Li S, Nur-Shafawati AR, Hatin WI, Ismail E, Mokhtar SS, et al. 2014. The population genomic landscape of human genetic structure, admixture history and local adaptation in Peninsular Malaysia. Hum Genet 133:1169-1185.

Depristo MA, Banks E, Poplin R, Garimella KV, Daly MJ. 2011. A framework for variation discovery and genotyping using next-generation DNA sequencing data. Nat Genet 43:491-498.

He YY, Wang XC, Jin PK, Zhao B, Fan X. 2009. Complexation of anthracene with folic acid studied by FTIR and UV spectroscopies. Spectrochim Acta A Mol Biomol Spectrosc 72:876-879.

Henn BM, Botigue LR, Gravel S, Wang W, Brisbin A, Byrnes JK, Fadhlaoui-Zid K, Zalloua PA, Moreno-Estrada A, Bertranpetit J, et al. 2012. Genomic ancestry of North Africans supports back-to-Africa migrations. PLoS Genet 8:e1002397.

Jablonski NG. 2004. The Evolution of Human Skin and Skin Color. Annual Review of Anthropology

33:585-623.
Jablonski NG, Chaplin G. 2000. The evolution of human skin coloration. J Hum Evol 39:57-106.
Jablonski NG, Chaplin G. 2010. Human skin pigmentation as an adaptation to UV radiation. Proc Natl Acad Sci U S A 107:8962–8968.
Juzeniene A, Stokke KT, Thune P, Moan J. 2010. Pilot study of folate status in healthy volunteers and in patients with psoriasis before and after UV exposure. J Photochem Photobiol B 101:111-116.
Ko AM, Chen CY, Fu Q, Delfin F, Li M, Chiu HL, Stoneking M, Ko YC. 2014. Early Austronesians: into and out of Taiwan. Am J Hum Genet 94:426-436.
Krishan G. 1993. Morpho-genetic studies among the Andaman and Micobar islanders. Anthropologie (1962-) 31:151-155.
Lazaridis I, Patterson N, Mittnik A, Renaud G, Mallick S, Kirsanow K, Sudmant PH, Schraiber JG, Castellano S, Lipson M, et al. 2014. Ancient human genomes suggest three ancestral populations for present-day Europeans. Nature 513:409-413.
Li H, Durbin R. 2010. Fast and accurate long-read alignment with Burrows-Wheeler transform. Bioinformatics 26:589-595.
Li H, Handsaker B, Wysoker A, Fennell T, Ruan J, Homer N, Marth G, Abecasis G, Durbin R, Genome Project Data Processing S. 2009. The Sequence Alignment/Map format and SAMtools. Bioinformatics 25:2078-2079.
Liu X, Yunus Y, Lu D, Aghakhanian F, Saw WY, Deng L, Ali M, Wang X, Ghazali F, Rahman TA, et al. 2015. Differential positive selection of malaria resistance genes in three indigenous populations of Peninsular Malaysia. Human Genetics 134:375-392.
Lu D, Lou H, Yuan K, Wang X, Wang Y, Zhang C, Lu Y, Yang X, Deng L, Zhou Y, et al. 2016. Ancestral origins and genetic history of Tibetan highlanders. Am J Hum Genet 99:580-594.
Lu J, Lou H, Fu R, Lu D, Zhang F, Wu Z, Zhang X, Li C, Fang B, Pu F, et al. 2017. Assessing genome-wide copy number variation in the Han Chinese population. J Med Genet 54:685-692.
Mallick S, Li H, Lipson M, Mathieson I, Gymrek M, Racimo F, Zhao M, Chennagiri N, Nordenfelt S, Tandon A, et al. 2016. The Simons Genome Diversity Project: 300 genomes from 142 diverse populations. Nature 538:201-206.
Manichaikul A, Mychaleckyj JC, Rich SS, Daly K, Sale M, Chen WM. 2010. Robust relationship inference in genome-wide association studies. Bioinformatics 26:2867-2873.
McEvoy BP, Powell JE, Goddard ME, Visscher PM. 2011. Human population dispersal "Out of Africa" estimated from linkage disequilibrium and allele frequencies of SNPs. Genome Res 21:821-829.
McKenna A, Hanna M, Banks E, Sivachenko A, Cibulskis K, Kernytsky A, Garimella K, Altshuler D, Gabriel S, Daly M, et al. 2010. The Genome Analysis Toolkit: a MapReduce framework for analyzing next-generation DNA sequencing data. Genome Res 20:1297-1303.
Mondal M, Casals F, Xu T, Dall'Olio GM, Pybus M, Netea MG, Comas D, Laayouni H, Li Q, Majumder PP, et al. 2016. Genomic analysis of Andamanese provides insights into ancient human migration into Asia and adaptation. Nat Genet 48:1066-1070.
Norton HL, Friedlaender JS, Merriwether DA, Koki G, Mgone CS, Shriver MD. 2006. Skin and hair pigmentation variation in Island Melanesia. Am J Phys Anthropol 130:254-268.
Pagani L, Lawson DJ, Jagoda E, Morseburg A, Eriksson A, Mitt M, Clemente F, Hudjashov G, DeGiorgio M, Saag L, et al. 2016. Genomic analyses inform on migration events during the

peopling of Eurasia. Nature 538:238-242.
Parra EJ, Kittles RA, Shriver MD. 2004. Implications of correlations between skin color and genetic ancestry for biomedical research. Nature Genetics 36:S54-S60.
Patin E, Siddle KJ, Laval G, Quach H, Harmant C, Becker N, Froment A, Regnault B, Lemee L, Gravel S, et al. 2014. The impact of agricultural emergence on the genetic history of African rainforest hunter-gatherers and agriculturalists. Nat Commun 5:3163.
Petersen DC, Libiger O, Tindall EA, Hardie RA, Hannick LI, Glashoff RH, Mukerji M, Indian Genome Variation C, Fernandez P, Haacke W, et al. 2013. Complex patterns of genomic admixture within southern Africa. PLoS Genet 9:e1003309.
Pugach I, Delfin F, Gunnarsdottir E, Kayser M, Stoneking M. 2013. Genome-wide data substantiate Holocene gene flow from India to Australia. Proc Natl Acad Sci U S A 110:1803-1808.
Purcell S, Neale B, Todd-Brown K, Thomas L, Ferreira MA, Bender D, Maller J, Sklar P, de Bakker PI, Daly MJ, et al. 2007. PLINK: a tool set for whole-genome association and population-based linkage analyses. Am J Hum Genet 81:559-575.
Rawofi L, Edwards M, Krithika S, Le P, Cha D, Yang Z, Ma Y, Wang J, Su B, Jin L, et al. 2017. Genome-wide association study of pigmentary traits (skin and iris color) in individuals of East Asian ancestry. PeerJ 5:e3951.
Reich D, Patterson N, Kircher M, Delfin F, Nandineni MR, Pugach I, Ko AM, Ko YC, Jinam TA, Phipps ME, et al. 2011. Denisova admixture and the first modern human dispersals into Southeast Asia and Oceania. Am J Hum Genet 89:516-528.
Rocha J. 2020. The Evolutionary History of Human Skin Pigmentation. J Mol Evol 88:77-87.
Schlebusch CM, Skoglund P, Sjodin P, Gattepaille LM, Hernandez D, Jay F, Li S, De Jongh M, Singleton A, Blum MG, et al. 2012. Genomic variation in seven Khoe-San groups reveals adaptation and complex African history. Science 338:374-379.
Štěrbová Z, Tureček P, Kleisner K. 2019. Consistency of mate choice in eye and hair colour: Testing possible mechanisms. Evolution and Human Behavior 40:74-81.
Supek F, Bosnjak M, Skunca N, Smuc T. 2011. REVIGO summarizes and visualizes long lists of gene ontology terms. PLoS One 6:e21800.
The 1000 Genomes Project Consortium. 2015. A global reference for human genetic variation. Nature 526:68-74.
